# Novel quantitative trait loci from an interspecific *Brassica rapa* derivative improve pod shatter resistance in *Brassica napus*

**DOI:** 10.3389/fpls.2023.1233996

**Published:** 2023-09-06

**Authors:** Harsh Raman, Rosy Raman, Niharika Sharma, Xiaobo Cui, Brett McVittie, Yu Qiu, Yuanyuan Zhang, Qiong Hu, Shengyi Liu, Nelson Gororo

**Affiliations:** ^1^ New South Wales (NSW) Department of Primary Industries, Wagga Wagga Agricultural Institute, Wagga Wagga, NSW, Australia; ^2^ New South Wales (NSW) Department of Primary Industries, Orange Agricultural Institute, Orange, NSW, Australia; ^3^ Oil Crops Research Institute, Chinese Academy of Agricultural Sciences, Wuhan, Hubei, China; ^4^ Nuseed Pty Ltd, Horsham, VIC, Australia

**Keywords:** pod shattering, domestication, genetic mapping, canola, genetic analysis, sequence variation

## Abstract

Pod shatter is a trait of agricultural relevance that ensures plants dehisce seeds in their native environment and has been subjected to domestication and selection for non-shattering types in several broadacre crops. However, pod shattering causes a significant yield reduction in canola (*Brassica napus* L.) crops. An interspecific breeding line BC95042 derived from a *B. rapa/B. napus* cross showed improved pod shatter resistance (up to 12-fold than a shatter-prone *B. napus* variety). To uncover the genetic basis and improve pod shatter resistance in new varieties, we analysed F_2_ and F_2:3_ derived populations from the cross between BC95042 and an advanced breeding line, BC95041, and genotyped with 15,498 DArTseq markers. Through genome scan, interval and inclusive composite interval mapping analyses, we identified seven quantitative trait loci (QTLs) associated with pod rupture energy, a measure for pod shatter resistance or pod strength, and they locate on A02, A03, A05, A09 and C01 chromosomes. Both parental lines contributed alleles for pod shatter resistance. We identified five pairs of significant epistatic QTLs for additive x additive, additive dominance and dominance x dominance interactions between A01/C01, A03/A07, A07/C03, A03/C03, and C01/C02 chromosomes for rupture energy. QTL effects on A03/A07 and A01/C01 were in the repulsion phase. Comparative mapping identified several candidate genes (*AG*, *ABI3*, *ARF3*, *BP1*, *CEL6*, *FIL, FUL*, *GA2OX2*, *IND*, *LATE*, *LEUNIG*, *MAGL15*, *RPL*, *QRT2*, *RGA*, *SPT* and *TCP10*) underlying main QTL and epistatic QTL interactions for pod shatter resistance. Three QTLs detected on A02, A03, and A09 were near the *FUL (FRUITFULL)* homologues *BnaA03g39820D* and *BnaA09g05500D*. Focusing on the *FUL*, we investigated putative motifs, sequence variants and the evolutionary rate of its homologues in 373 resequenced *B. napus* accessions of interest. *BnaA09g05500D* is subjected to purifying selection as it had a low Ka/Ks ratio compared to other *FUL* homologues in *B. napus.* This study provides a valuable resource for genetic improvement for yield through an understanding of the genetic mechanism controlling pod shatter resistance in *Brassica* species.

## 1 Introduction

**Figure 1:**
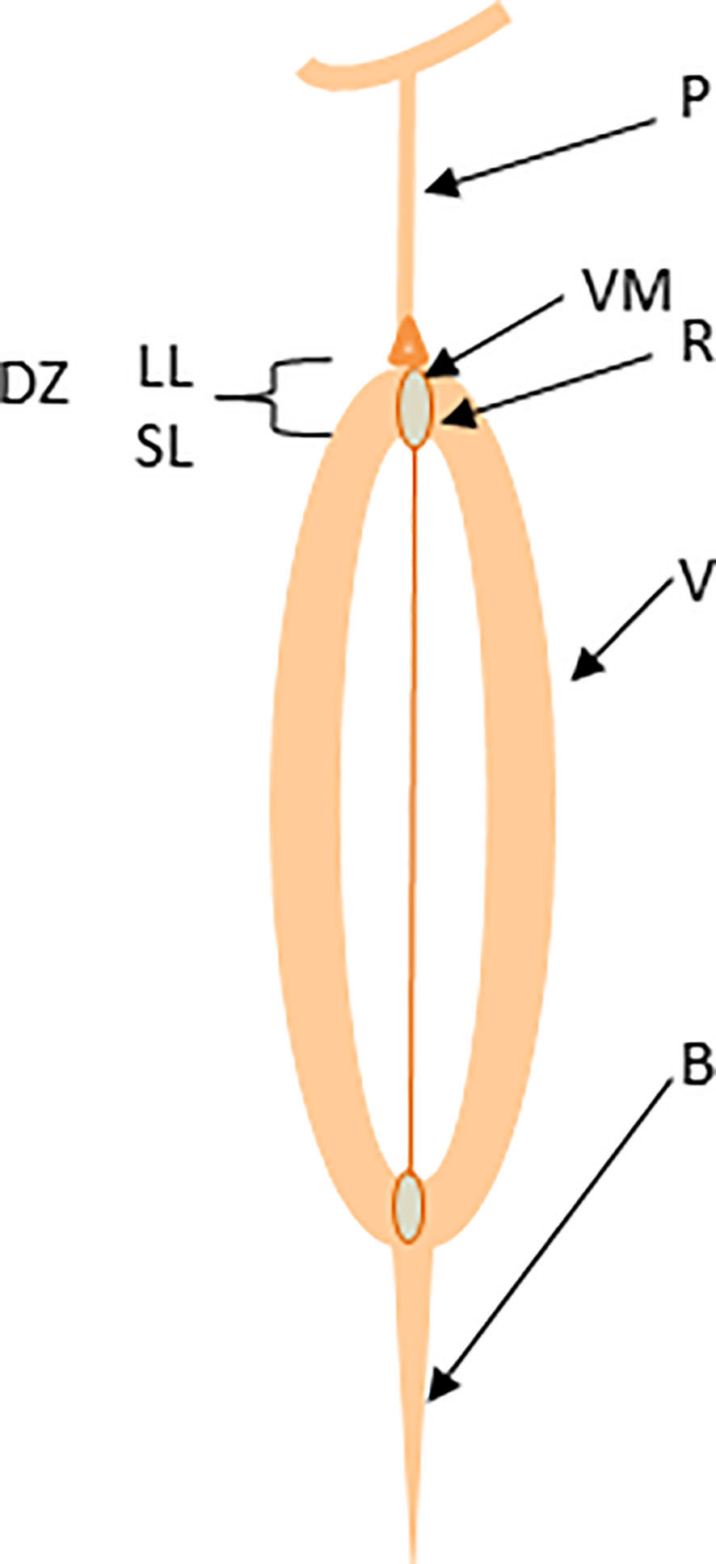
Schematic representation of canola pod structure. The valve (V), dehiscence zone (DZ), separation layer (SL), lignification layer (LL), valve margin (VM), replum (R), and beak (B).

Plants have evolved vivid mechanisms for survival and fitness across various ecological niches. In the wild, plants dehisce their fruits and disperse seeds to ensure the multiplication and adaptation of their progenies and confront challenges posed by climatic and ecological vagaries. Seeds of the *Brassicaceae* family members are enclosed in a silique (pod), which consists of two congenitally fused carpels (valves); each is separated with a thin layer called a pseudo-septum or replum (**Figure 1**) (Bowman et al., 1999). Both valves and replum are differentiated with valve margins where pod dehiscence and seed abscission occur via pod drop and seed shattering, possibly by similar molecular mechanisms (Balanzà et al., 2016). Pod drop – a phenomenon where a whole fruit (silique) drops on the ground, is a common problem in some canola production regions, particularly Canada. As the pod matures physiologically, valves detach from the replum, resulting in pod dehiscence (**Figure S1A**) and the seeds attached to the replum with a funiculus fall to the ground (**Figure S1C**). Pod dehiscence occurs via the dehiscence zone formation at the valve margins by two layers: a lignification layer of 1-2 thick and rigid cells and the separation (also called abscission) layer of iso-diametrically shaped cells, separating the valve from the replum (Spence et al., 1996; Rajani and Sundaresan, 2001; Dinneny and Yanofsky, 2005). At maturity, cells in the separation layer degrade by polygalacturonase, cellulase, and mannanase enzymes (Ogawa et al., 2009). Shattering occurs when the abscission force becomes more significant than the binding force of the pod valve (Lee et al., 2017). External influences such as wind velocity, machinery, and high temperatures further escalate pod shattering in brassicas.

Molecular mechanisms underlying pod dehiscence are well-dissected in a model plant, *Arabidopsis thaliana* - a distant relative of *Brassica napus* L. At least thirteen genes that are responsible for pod dehiscence in Arabidopsis have been identified, such as MADS-box genes: *SHATTERPROOF1* (*SHP1), SHATTERPROOF*2 *(SHP2*) and *FRUITFULL* (*FUL*); Basic-loop-helix genes: *INDEHISCENT* (*IND*), *ALCATRAZ* (*ALC*) and *SPATULA (SPT); REPLUMLESS* (*RPL*) and *APETALA2* (*AP2*), *ARABIDOPSIS DEHISCENCE ZONE POLYGALACTUROSE1* (*ADPG1*), ADPG2, a C2H2 zinc finger transcription factors *JAGGED* (*JAG*) and BnLATE FLOWERING (*BnLATE)*; NAC SECONDARY WALL THICKENING PROMOTING FACTOR1 (*NST1*), ENDO-BETA-*MANNANASE*7 (*MAN7*), and *CELLULASE6* (Ferrandiz et al., 2000; Liiljegren et al., 2000; Rajani and Sundaresan, 2001; Roeder et al., 2003; Sorefan et al., 2009; He et al., 2018; Li et al., 2021). Different genes involved in auxin, gibberellin and cytokinin biosynthesis also regulate pod development and dehiscence (Sorefan et al., 2009; Arnaud et al., 2010; Marsch-Martínez et al., 2012).

Canola, the second most crucial oilseed crop after soybean, contributes about 13-16% of global vegetable oil production. The allotetraploid canola genome (2*n* = 4× = 38, genome AACC) originated about 7,500 years ago via ancient hybridisation events between two diploid progenitors *Brassica* species, *B. rapa* (2*n* = 2× = 20, AA genome) and *B. oleracea* (2*n* = 2× = 18, CC genome) (Chalhoub et al., 2014; Lu et al., 2019). However, seed shattering (commonly referred to as pod-shattering) is a universal constraint in canola production, and in the literature, none of the domesticated accessions of *B. napus* is reported to be ‘completely’ resistant to pod shattering. Generally, canola pods are highly sensitive to pre-mature shattering, significantly reducing yield. The seed loss varies from 8 to 70% across environments depending on genotypic attributes (canopy architecture, resistance to lodging and diseases), method of harvesting (windrow/direct heading), and time of harvesting (early, optimal time vs late) and environmental conditions at the time of harvest (MacLeod, 1981; Price et al., 1996; Child et al., 1998; Vera et al., 2007; de la Pasture, 2018). Shattered seeds grow in the field at a much higher rate (60x) than those sowed initially (**Figure S1**) and become a weed in the next crop; hence must be controlled (Wang et al., 2007).

To overcome pod-shattering, the majority of broadacre canola varieties are harvested by windrowing/swathing - a practice of cutting plants at physiological maturity (50 to 60% seed colour change from green to dark brown, red or black) and leaving them in the field before threshing with a combine harvester. This practice can also lead to significant losses from seed shattering, mainly when not accomplished at the ‘right’ time. The window for windrowing is often small and subjected to labour and combined harvester availability and congenial weather conditions. High temperatures, high-velocity winds, rainfall, and hailstorm events significantly impact canola seed yield and oil content. High yield is essential for meeting global demands for healthy vegetable oil, protein for animal feed, and canola growers for return on their investment.

Understanding the genetic determinants and novel alleles underlying this domestication trait would provide an improved genetics-based solution to reduce yield loss in *B. napus*. The functionality of some of Arabidopsis pod dehiscence genes has also been demonstrated in *Brassica* species via overexpression, RNAi, gene editing, and induced mutation studies (Ostergaard et al., 2006; Kord et al., 2015; Lawrenson et al., 2015; Braatz et al., 2018a; Braatz et al., 2018b; Stephenson et al., 2019; Li et al., 2021). Recently, it has also been shown that miR319-targeted *TEOSINTE BRANCHED 1*, *CYCLOIDEA*, and *PROFEERATIN CELL NUCLEAR ANTIGEN BINDING FACTOR* (*TCPs*) inhibit pod elongation and dehiscence via regulation of *FUL* expression in *A. thaliana* and *B. napus* (Cao et al., 2022). Although the network of pod dehiscence genes has been investigated in Arabidopsis, their expression level has not been fine-tuned in commercial canola varieties with genetic modification approaches, except in POD GURAD varieties where TILLING has been deployed only in the BASF canola breeding program (Laga et al., 2008). In fact, ectopic (over-) expression of *FUL* and *SHP* genes led to indehiscent pods due to the non-lignification of cells between the valve and replum and the absence of dehiscence zone formation (Ferrandiz et al., 2000; Liiljegren et al., 2000; Ostergaard et al., 2006).

Previous research has shown a limited range of genetic variation for pod shatter resistance in *B. napus* (Morgan et al., 2007; Raman et al., 2014). However, a wide range of genetic variation for pod shattering is observed in diploid and amphidiploid species of *Brassica*, such as *B. rapa, B. juncea* (2*n* = 4× = 36, AABB), and *B. carinata* (2*n* = 4× = 34, BBCC) (Kadkol et al., 1984; Kadkol et al., 1985; Raman et al., 2017). In a previous study, Raman et al. (2014) reported that pod shatter resistance could improve up to 12-fold in a shatter-prone variety of *B. napus* via the introgression of resistant alleles from *B. rapa*. To uncover the genetic basis underlying seed shattering in this interspecific source, we investigated an F_2_ mapping population and its F_2:3_ progenies derived from a cross between *B. napus* (BC95041) and *B. rapa*/*B. napus* (BC95042). We further identified epistatic quantitative trait loci (QTLs) for additive × additive, additive dominance, and dominance × dominance interactions. Candidate genes and their sequence variants in parental lines underlying QTL regions for pod shatter resistance were identified, which could regulate variation in pod shatter resistance.

## 2 Materials and methods

### 2.1 Construction of mapping population

An interspecific line derived *B. rapa/B. napus* with the highest pod rupture energy (RE), BC95042 (shatter resistant with high RE (Raman et al., 2014)) was crossed with the advanced breeding lines of *B. napus*, BLN3303 (BC95041, maternal parent, shatter prone with low RE). This study utilised an F_2_ population comprising 203 individuals generated from the self-pollination of a single F_1_ cross from BC95041/BC94042. Each F_2_ line was selfed to generate an F_2:3_ population for confirming phenotypes.

### 2.2 Evaluation for pod shatter resistance

The two parental lines and their F_2_ population of 203 plants were grown in 2021 in white plastic pots (Garden City Plastics, NSW, Australia)) under birdcage conditions at the Wagga Wagga Agricultural Institute, New South Wales, Australia. The cultivation of canola plants followed standard management practices. Plants were watered thrice per week, fertilised weekly using in-line liquid fertilisers, and protected from blackleg and sclerotinia diseases by applications of Prosaro^®^ 420 SC and Aviator fungicides (Bayer Crop Sciences, Australia) and aphids using chemicals recommended in Australia. Day to flowering was recorded daily for each F_2_ plant. To avoid outcrossing and get pure F_3_ progenies, all F_2_ plants were bagged with perforated pollination bags before flower initiation, leaving the primary stem out for the natural pod development for shatter testing. Ten pods were collected from each line at maturity (BBCH scale 95) in the 50 mL plastic tubes containing a silica sachet, as detailed in our previous study (Raman et al., 2014). Pods were desiccated in a dehydrator (G. T. D. Pty. Ltd., Australia) at 40°C for 48 hours to reduce variation due to moisture content and further tested for variation in pod rupture energy. For validation, 40 F_2:3_ families (20 high rupture energy and 20 low rupture energy) and parents were grown in pots in 2016 under birdcage conditions and tested with a pendulum test described earlier (Raman et al., 2014). The phenotypic means for each genotype were used for further genetic analysis. A pair-wise correlation between rupture energy and pod length in F_2_ and F_2:3_ populations was calculated. The rupture energy of five pods of each F_2_ plant was averaged and used for QTL analysis.

### 2.3 DNA isolation and genotyping

Young leaf tissue of the field-grown plants was collected from each line in a 96-well format. The tissue was frozen immediately and kept at - 80°C until used for DNA isolation. Tissue was ground in liquid nitrogen and extracted for DNA using a method described by Raman et al. (2005). DNA concentration was determined by a Qubit fluorometer and Qubit dsDNA broad-range assay kit according to the manufacturer’s recommendation. DNA quality was checked on the Tris-Acetate-EDTA buffered 0.8% agarose gel. The F_2_ population and parental lines were genotyped with the genotyping-by-sequencing-based DArTseq marker approach (Raman et al., 2014) using the HiSeq 2500 system (Illumina, USA) at the DArT P/L, University of Canberra, Bruce, Australia. We considered only high-quality DArTseq markers, which included SNPs (single nucleotide polymorphism) and *in-sillco* presence-absence markers, having BLAST alignments (E-value: 5e^-5^) and minimum sequence identity of 90% with the reference *B. napus* cv. Darmor-*bzh* v 4.1.

### 2.4 Map construction and QTL identification for pod shatter resistance

The linkage map of the F_2_ population was constructed using DArT P/L’s OCD MAPPING program (Petroli et al., 2012), as described previously (Raman et al., 2017). The association between markers and rupture energy was tested using linear marker regression, Fisher’s exact test, and the *X^2^* test. We applied the additive, dominant and recessive models and full scan permutation with 1000 iterations for the genome scan. Haplotype blocks (HB) were detected using 0.98 upper confidence and 0.7 lower bound recombination value at threshold 0.01, Expectation maximization algorithm (EM) iteration 1,000 and EM convergence tolerance value of 0.00010 (Gabriel et al., 2002). *P* values for haplotyping association test were determined using 10,000 iterated permutations in the SVS package (Golden Helix, Bozeman, USA). We used binary data of contrasting 141 F_2_ phenotypes for resistance or sensitivity to shattering (**Table S5a**) for haplotype analysis. Manhattan plots were generated in the SVS package (Golden Helix, Bozeman, USA).

QTL mapping was performed by single interval mapping (IM), inclusive composite interval mapping (ICIM-ADD) of additive and dominant QTL, and inclusive composite interval mapping of epistatic QTL (ICIM-EPI) functions implemented in the QTL IciMapping v4.1 (www.isbreeding.net). The threshold logarithm of odds (LOD) value was determined by a permutation test involving 1,000 runs at a significance level of *P* = 0.05. Threshold *P* values for ICIM and IM for rupture energy were 3.07 and 3.25, respectively. While for pod length, threshold *P* values for ICIM and IM are 2.66 and 1.78, respectively. QTLs having LOD values more than the estimated threshold were declared as significant. LOD score greater than 2.5 but less than estimated threshold *P* values were termed suggestive QTL. The phenotypic variance explained (% PVE) and the additive effects of QTLs were directly derived from the QTL analysis outputs files. For digenic epistatic QTL interactions, LOD threshold values for each trait were estimated after 1,000 permutations using a type I error = 0.05. Epistatic effect QTLs were analysed using ICIM-EPI at the threshold LOD 4.87. Favorable parental alleles that enhance the trait expression were identified using an additive effect’s direction (+ and -ve).

### 2.5 Alignment of markers with the Brassica reference genomes

The physical map positions of significant markers associated with pod shatter resistance were obtained using the reference *B. napus cv* Darmor*-bzh* genome by BlastN (Altschul et al., 1990) searches, as detailed in Raman et al. (2014). We also used the BnaOmics platform (https://bnaomics.ocri-genomics.net/) that integrates pan-genome and multi-omics data of *B. napus* (Cui et al., 2023) to search candidate genes. The only single top hit with the cutt-of E value of 1E^-5^ was considered for identifying syntenic region underlying candidate genes. *B. napus* annotated genes which were mapped within the marker intervals with ICIM/ICIM-EPI, were assumed candidate genes. The candidates that map within 500 kb from the significant markers identified with genome scan approaches were also identified. Genes involved in the pod shatter trait of *Arabidopsis* (**Table S14**) were used to search the corresponding copies in *B. napus*, with an e-value of 1e^-10^.

### 2.6 Identifying *FUL* homologues in *B. napus* based on homology to *ATFUL* (AT5G60910)

*Arabidopsis thaliana* genic and protein sequences of AT5G60910 from the Arabidopsis Information Resource (TAIR) were used to search the homologues in *B. napus* using TBLASTN and BLASTP (*B. napus* cv. Darmor-*bzh* genome, versions 4.1; http://www.genoscope.cns.fr, and the pan-genome) (Cui et al., 2023).

### 2.7 Phylogenetic relationship and Ka/Ks ratios

**Figure 2:**
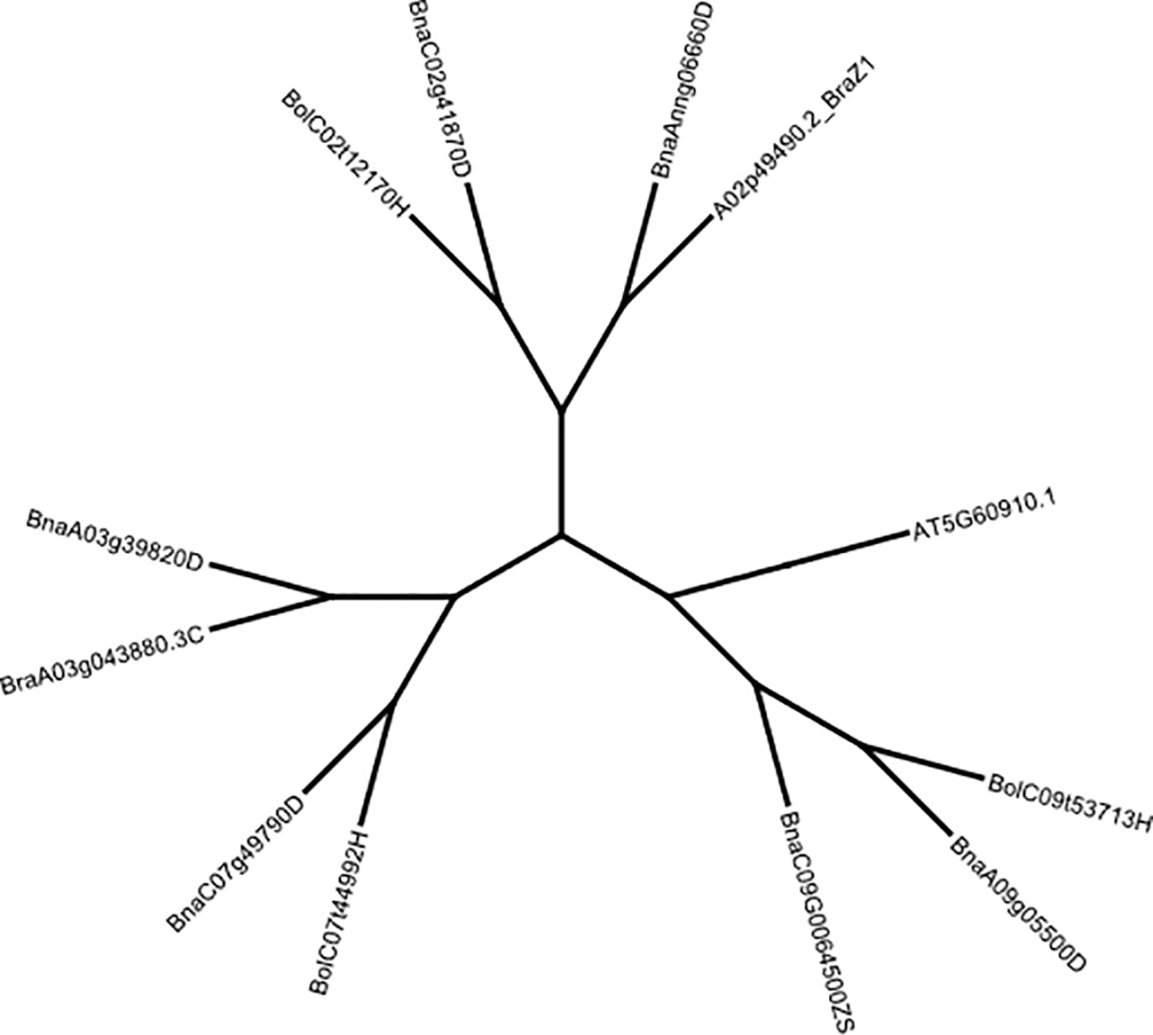
Neighbour-joining tree showing the grouping of *B. rapa*, *B oleracea* and *B. napus FUL* copies using Jukes-Cantor distance and Blosum62 cost matrices implemented in Geneious Prime. The *FUL* gene of *A. thaliana* (AT5G60910, TAIR) was used as an outgroup. Multiple sequence alignments were also carried-out for the *FUL* homologues. FUL protein sequences were retrieved from the BRAD database (www.brassicadb.cn, Accessed 18 April 2023).

We used the Geneious tree builder pipeline to generate a Neighbour-Joining phylogenetic tree of DNA sequences from *B. rapa*, *B. oleracea* and *B. napus* for *FUL* (**Figure 2**) and *FUL-Like* genes (**Figure S5**). Sequences were aligned with global alignment with free end gaps, Blosum62 cost matrix, and Jukes-Cantor genetic distance model, implemented in the Geneious prime package (https://www.geneious.com). *A. thaliana FUL* gene was used as an outgroup to verify functional divergence. The synonymous substitution rate (Ks), non-synonymous substitution rate (Ka), and Ka/Ks ratio were calculated with SNPGenie (https://github.com/chasewnelson/SNPGenie).

### 2.8 Gene structure and motif conserved domains and *cis*-acting elements identification of *FUL* homologues

The intron-exon distribution of *FUL* genes was obtained from genome annotation files from the online resources described above and confirmed using sequence analysis with *AtFUL*. Multiple sequence alignment of protein sequences was performed with ClustalX 2.0 (http://www.custal.org/clustal2/) and implemented in the BioEdit package to visualise functional variation in the *FUL* genes. Conserved domains in the FUL were predicted using the NCBI Conserved Domain Database (http://www.ncbi.nih.gov/cdd) at E-value <0.001. Analysis of 5Kb upstream sequences of five *FUL* homologues for locating known motifs in the cis-acting regulatory elements was conducted using SIGNALSCAN program in Plant cis-Regulatory DNA Elements (PLACE, https://www.dna.affrc.go.jp/PLACE/?action=newplace). The number of motifs identified for each type were counted, and their roles were described (https://www.dna.affrc.go.jp/PLACE/place_seq.shtml). Also, the same dataset (5Kb upstream sequences of *FUL* homologues) was investigated for the presence of any novel motifs (sequence pattern that repeatedly occurs in a group of related protein or DNA sequences) using MEME (Multiple EM for Motif Elicitation, https://meme-suite.org/meme/tools/meme).

### 2.9 Microscopic analysis of pod anatomy

Anatomical features of valve margins from pods of parental lines were collected 35 to 40 days after anthesis. Hand sections were prepared from the middle of the pod, where the replum was narrow. Fresh sections were observed for autofluorescence using a fluorescence microscope. Photographs were taken using a Zeiss Axiphot microscope fitted with a Sony Cyber-shot digital camera.

## 3 Results

### 3.1 Inheritance of pod shatter resistance

We evaluated 203 F_2_ lines derived from a cross between the *B. napus* line BLN3343-C00402 (maternal parent, NBGIP accession BC95041, shattering type) and interspecific line BC95042 (paternal parent derived from *B. rapa/B. napus*, resistant to pod shattering, Raman et al., 2014) using the pendulum test to investigate the genetic inheritance and genetic determinants underlying pod-shattering resistance. Herein, we implemented the pendulum test to detect genetic variation in rupture energy - a measure of pod strength/resistance to shattering (Kadkol et al., 1984; Kadkol et al., 1986; Liu et al., 1994; Raman et al., 2014). The interspecific line, BC95042, required a higher level of force to break up the pod and release seed; therefore, it had a higher value for rupture energy than the maternal line BC95041.

**Figure 3:**
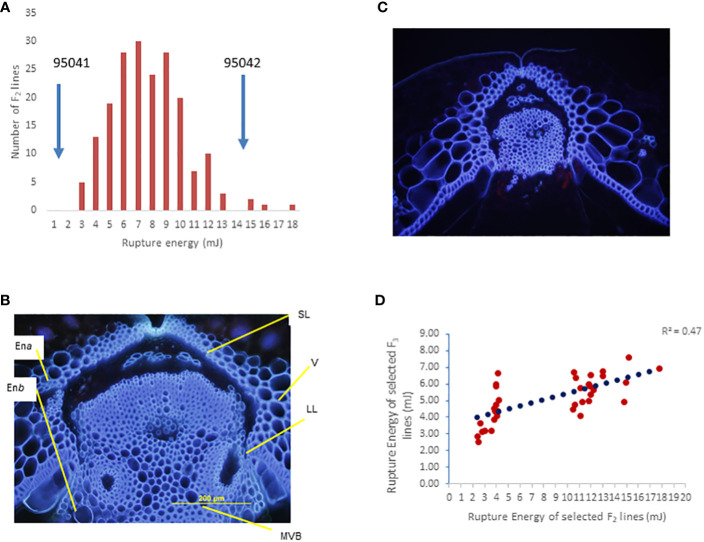
Genetic analysis of the BC95041 (shatter prone)/BC95042 (shatter resistant) F_2_ population for pod shatter resistance. **(A)** Frequency distribution of rupture energy (RE) scores in a segregation population containing 179 individuals. Solid arrows indicate the average RE scores of the maternal line BC95041 and the paternal interspecific line 95042. **(B)** Cross section of developing pods showing well-developed dehiscence zone/abscission layer (DZ) in BC95041, whereas BC95042 shows limited DZ differentiation. The dehiscence zone: DZ, valve: V, the main vascular bundle of replum: MVB, the two endocarp layers, endocarp a: En*a*, and endocarp b: En*b* are shown. **(C)** Arrowheads indicate a lack of complete cell separation in the pod shatter-prone line. **(D)** Relationship `of pod rupture energy scores between F_2_ and F_3_ individuals.

The F_2_ population derived from a single F_1_ plant showed a continuous distribution of rupture energy scores, ranging from 2.32 mJ to 17.76 mJ) (**Figure 3A**). We observe that both pod valves separate length-wise (vertically) under field conditions (**Figure S1A**). This shattering pattern differs from pod drop, which often occurs in related species of *Brassica*, such as *Raphanus raphanistrum subsp. sativus* (L.) (**Figure S1B**). Microscopic analysis revealed that the dehiscence zone is well-differentiated in shatter-prone parental lines of the mapping population BC95041 compared to pod shatter-resistant parental lines (BC95042). Interspecific line BC95042 required high energy to rupture the pod (threshing) than the shatter-prone line BC95041 (**Figure 3A**). In the resistant parental line, there was less lignification of cells near the dehiscence zone and a less conspicuous distinction between lignified and separation layer from the replum compared to shatter-prone lines (**Figures 3B, C**). These observations suggest that the pod shatter resistance genes play an essential role in the dehiscence zone differentiating and subsequent seed dispersal (Liiljegren et al., 2000). To verify the rupture energy scores of the F_2_ lines, we raised a subset of 40 F_2:3_ progenies representing extreme phenotypes (the top 20 and bottom 20 F_2_ lines based on their pod energy scores) under natural field conditions. A positive correlation (*r* = 0.7) between the rupture energy scores of F_2_ plants and their F_2:3_ progenies (**Figure 3D**) indicates that rupture energy scores are reliable and suitable for genetic analysis.

### 3.2 Multiple loci associated with resistance to pod shatter

**Figure 4:**
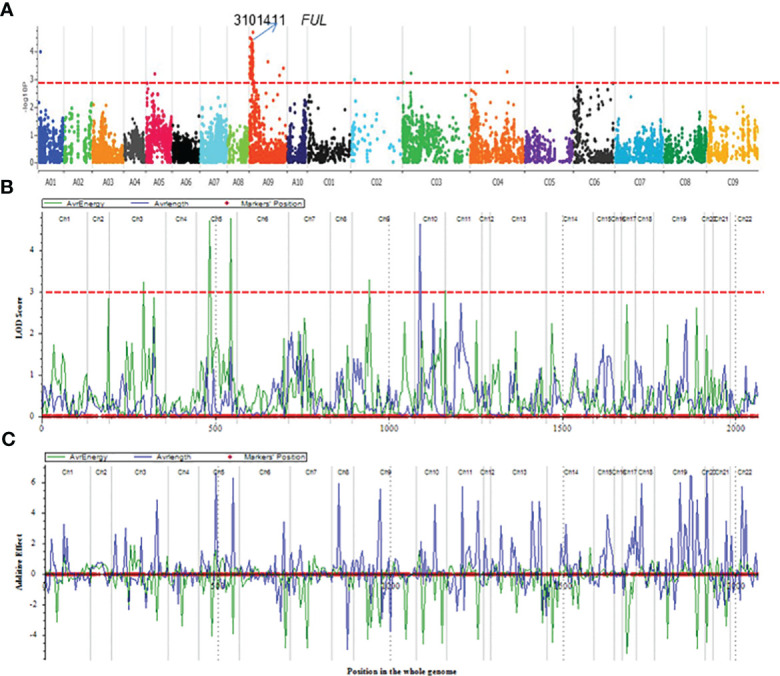
QTL mapping for pod shatter resistance measured as rupture energy (mj) by the pendulum test and pod length in the F_2:3_ population derived from BC95041/BC95042. **(A)** Manhattan plots showing genomic regions associated with resistance to pod shatter: significant regions are labelled. **(B)** Gene scan showing a single QTL on chromosome A09 for pod shatter resistance in an F_2_ population derived from a cross between BC95041 and BC95042. Significant QTL having a LOD score of 4 are shown by the dashed line (in blue colour). Pod shatter resistance was evaluated under birdcage conditions at Wagga Wagga, Australia and tested for rupture energy using a pendulum. **(C)** Allelic effects estimated by CIM approach. Linkage groups: Ch1-Ch10 relate to chromosomes A1-A10, Ch11-Ch14 to C01-C04; Ch15-16 to C05, Ch17-18 to C06, Ch19 to C07, Ch20-21 to C08 and Ch22 to C09.

Using the DArTseq technology (Raman et al., 2014), a total of 26,002 high-quality SNPs (single nucleotide polymorphism) and *in-sillco* presence-absence markers, which showed (i) polymorphism between the parents and (ii) segregation in a mapping population, were used. We constructed a genetic linkage map spanning a total length of 2117.53 cM, with an average interval of 7.32 cM. The length of the chromosomes (linkage groups) ranged from 22.25 (C02) to 179.81 cM (A09). The marker density of the linkage groups ranged from 3.61 (A02) to 10.15 (A10). On average, 80.51% of markers were anchored to the 19 linkage groups, representing the A^n^ and C^n^ subgenomes of the reference *B. napus* cv. Darmor-*bzh* genome (**Table 1**). Using a genetic framework map based on 15,498 DArTseq markers (**Table S1**), we identified and located the significant QTLs conferring resistance to pod shatter on the *B. napus* genome. Different algorithms were used to identify robust associations for breeding use. Linear regression analysis using an additive model revealed that the top 99 markers mapped on chromosomes A01, A05, A09, C03 and C04 have a significant association (LOD ≥3.00) with resistance to pod shatter (**Figure 4A**, **Table S2A**). Of them, the top 16 markers were localised on A09 within 4.59 to 21.47 cM, and *in-silico* DArTseq marker 3101411 showed the most significant association (-*log*_10_ *P* = 5.16) with resistance to pod shatter (**Supplementary Table S2B**). This marker showed a complete linkage with 15 other markers (**Table S2C**). Haplotype-based association test was conducted to detect the association between observed variations of pod shatter and marker haplotypes rather than single SNPs using the SVS package. We detected 677 haplotype blocks (HB, **Supplementary Table S3A**) following parameters described by Gabriel et al. (2002). Two markers in HB 303 on A09 detected the most significant association for pod shatter resistance with logistic regression (**Table S3B**). Haplotype trend regression revealed that HB308 (delimited with 3105829|F|0-8:C>G-8:C>G, 5121480|F|0-11:T>C-11:T>C, 3074795|F|0-19:G>T-19:G>T, 5050199|F|0-8:T>C-8:T>C markers, followed by HB309 with 3146480|F|0-46:A>G-46:A>G was the most significantly associated with pod rupture energy in the BC95041/BC95042 population (**Table S4**).

**Table 1:** Linkage map showing genetic distance, distribution and distance (cM) of DArTseq markers in the F_2_ population from BC95041/BC95042.

Chromosome	Mapped markers (No)	Total length (cM)	Average marker density	Markers mapped on AC genome	Markers mapped on the physical *B. napu*s cv Darmor-*bzh* genome (%)
A01	1060	136.94	7.74	224	78.87
A02	246	68.23	3.61	45	81.71
A03	1050	165.86	6.33	234	77.71
A04	757	89.89	8.42	158	79.13
A05	1020	118.09	8.64	226	77.84
A06	1465	149.78	9.78	268	81.71
A07	892	121.99	7.31	166	81.39
A08	618	64.91	9.52	113	81.72
A09	1481	179.81	8.24	284	80.82
A10	902	88.83	10.15	170	81.15
Total A subgenome	9491	1184.34	8.01	1888	80.11
C1	492	106.23	4.63	106	78.46
C2	83	22.25	3.73	6	92.77
C3	1214	174.60	6.95	226	81.38
C4	984	137.40	7.16	230	76.63
C5	427	88.19	4.84	65	84.78
C6	524	96.52	5.43	101	80.73
C7	1012	148.07	6.83	171	83.10
C8	626	78.63	7.96	104	83.39
C9	645	81.30	7.93	123	80.93
Total C subgenome	6007	933.19	6.44	1132	81.16
Total A and C genomes	15498	2117.53	7.32	3020	80.51

We further detected QTLs associated with rupture energy and pod length using the simple interval mapping (IM) and composite interval mapping (CIM) approaches using the ICIM package. Five to seven significant QTLs for rupture energy were detected on chromosomes A03, A05 and A09 and C01 with IM and CIM (**Table 2**, **Figure 4B**). Three consistent QTLs were localised to the same genomic regions on chromosomes A02 and A05 across the analytical methods (**Table 2**). LOD scores of QTLs ranged from 2.8 to 4.77 and accounted proportion of variance explained (PVE) from 6.29% to 20.80% (**Table 2**). QTLs displayed both additive and dominant effects. Both parental lines contributed alleles for pod shatter resistance (**Figure 4C**). However, the interspecific paternal line BC95042 showed higher allelic effects (more than 2 folds) than the maternal *B. napus* line BC95041.

**Table 2:** Quantitative Trait Loci (QTLs) associated with pod shatter resistance measured as average rupture energy with the pendulum test.

Mapping approach	Chromosomal location	DArTseq Marker	Physical position on Darmor-bzh v4.1	DArTseq Marker	Physical position on Darmor-bzh v4.1	LOD	PVE (%)	Additive effect	Dominant effect
Composite interval mapping of additive QTL									
	***A02***	*3129258|F|0-32:G>A-32:G>A	***23443447***	***4335059|F|0-41:T>C-41:T>C***	***24434057***	***2.84***	***9.42***	***0.07***	***2.05***
	*A03*	*3095606|F|0-36:A>T-36:A>T*	*14823303*	*3100670|F|0-31:A>G-31:A>G	*12171871 on chrAnn_random*	*3.24*	*20.80*	*-1.57*	*-1.50*
	*A03*	*5048176|F|0-11:C>T-11:C>T*	*19780019*	*3100404|F|0-57:G>T-57:G>T	*21580461*	*2.87*	*19.25*	*-3.02*	*-3.14*
	***A05***	***3089648|F|0-11:G>A-11:G>A***	***5420258***	*3089864|F|0-22:T>C-22:T>C	***5947676***	***4.71***	***13.06***	***-4.04***	***-5.12***
	***A05***	***4116883|F|0-10:C>T-10:C>T***	***19860330***	*3101784|F|0-53:A>G-53:A>G	***20067798***	***4.77***	***16.30***	***-3.89***	***-3.79***
	*A09*	*3082931|F|0-57:C>T-57:C>T*	*6081612*	*4167404|F|0-5:A>G-5:A>G*	*8328617*	*3.29*	*15.72*	*-3.51*	*-3.67*
	*C01*	*3101048|F|0-47:C>T-47:C>T*	*1404201*	*4110108|F|0-53:C>T-53:C>T*	*1469395*	*3.03*	*6.29*	*-1.05*	*0.04*
Single Interval mapping of additive QTL									
	***A02***	*3129258|F|0-32:G>A-32:G>A	***23443447***	***4335059|F|0-41:T>C-41:T>C***	***24434057***	***2.93***	***12.04***	***-0.17***	***2.30***
	***A05***	***3089648|F|0-11:G>A-11:G>A***	***5420258***	*3089864|F|0-22:T>C-22:T>C	***5947676***	***3.69***	***14.07***	***-4.28***	***-5.32***
	***A05***	***4116883|F|0-10:C>T-10:C>T***	***19860330***	*3101784|F|0-53:A>G-53:A>G	***20067798***	***3.83***	***18.51***	***-4.15***	***-4.36***
	*A09*	*5050053|F|0-9:T>G-9:T>G*	*1798316*	*5121480|F|0-11:T>C-11:T>C*	*4340953*	*2.91*	*9.22*	*1.17*	*0.49*
	*A09*	*5049291|F|0-34:G>A-34:G>A*	*2530510*	*3140648|F|0-36:T>C-36:T>C*	*2767343*	*2.80*	*17.72*	*1.78*	*-0.05*

DArTseq markers were binned, and DArTseq SNPs were used for QTL analysis. The logarithm of the odds (LOD) scores, additive effects, and the proportion of phenotypic variance (PVE) were estimated using the ICIM package. Permutation Loci detected across Composite Interval (ICIM) and simple interval mapping (IM) were in bold. *Distance, based on cosegregating loci as linked marker did not return a significant hit.

To investigate the major genetic determinants controlling rupture energy, we binned pod shatter variation scores into two discrete categories, resistant (1, rupture energy: 2.32 to 6.94 mJ) and susceptible (0, rupture energy: 7.0 to 17.76 mJ) phenotypes, in conjunction with the seven highly significant markers (**Supplementary Table S5A**) and performed haplotype analysis to determine trait-marker association. The chi-squared analysis supported the presence of a single shatter resistance gene in BnF_2_ (*χ^2^*_3:1 =_ 0.17, with 1 degree of freedom, Two-tailed *P* value = 0.90). The HB 309 (defined by 15 SNPs: 3146480|F|0-46:A>G-46:A>G, 3096696|F|0-28:T>C-28:T>C, 3101752|F|0-29:C>T-29:C>T, 3159673|F|0-15:T>G-15:T>G, 5818650|F|0-5:C>T-5:C>T, 7250077|F|0-9:G>A-9:G>A, 3076890|F|0-52:A>T-52:A>T, 3079266|F|0-41:T>A-41:T>A, 3113543|F|0-40:A>C-40:A>C, 5121412|F|0-9:A>G-9:A>G, 5120748|F|0-29:G>C-29:G>C, 7249512|F|0-32:T>C-32:T>C, 3076528|F|0-55:T>C-55:T>C, 3077272|F|0-18:C>T-18:C>T, 3081487|F|0-26:C>T-26:C>T) revealed the most significant marker association with pod shatter resistance (*χ^2^ -log*_10_P: 9.99, **Supplementary Table S5B**) on chromosome A09. No other significant association was detected on *B. napus* chromosomes. Significantly associated markers detected on A09 showed collinearity between genetic and physical maps (**Figure S2A**). Different analytic methods revealed at least one significant locus on chromosome A09 that conditions variation in pod shatter resistance in the BC95041/BC95042 population. Mendelisation of quantitative variation revealed the limitation of identifying significant QTLs for trait variation (**Tables 2**, **3**).

**Table 3:** Epistatic Quantitative Trait Loci (QTL) associated with pod shatter resistance measured as average rupture energy with the pendulum test.

Chromosome	LeftMarker1	Physical position on Darmor-bzh	RightMarker1	Physical position on Darmor-*bzh*	Chromosome	LeftMarker2	Physical position on Darmor-bzh	RightMarker2	Physical position on Darmor-bzh	LOD	PVE (%)	Add1	Add2	Dom1	Dom2	AddbyDom1	AddbyDom2	DombyAdd1	DombyAdd2
A01	4110587|F|0-9:C>G-9:C>G	2276310	3132222|F|0-58:T>C-58:T>C (A1 random)	155773	**C01**	**3101048|F|0-47:C>T-47:C>T**	**1713593**	4110108|F|0-53:C>T-53:C>T	**1644880**	5.44	17.45	2.39	-3.34	-3.98	-2.48	-2.55	-2.51	3.23	3.82
A03	5148873|F|0-19:G>A-19:G>A	5666135	4118427|F|0-10:A>G-10:A>G	6024022	C03	4121078|F|0-63:C>T-63:C>T	12585496	3141033|F|0-28:T>C-28:T>C	13581980	4.87	28.44	-0.46	-0.36	0.46	1.59	0.48	-2.75	0.50	-2.36
**A03**	*3100404|F|0-57:G>T-57:G>T	**14324688**	**5048176|F|0-11:C>T-11:C>T**	**21730375**	**A07**	**5029215|F|0-26:C>T-26:C>T**	**2562779**	*3078953|F|0-62:C>T-62:C>T	**2646233**	5.27	17.00	0.88	-0.77	-1.17	-0.84	-1.27	-1.43	0.99	0.51
**A07**	**5029215|F|0-26:C>T-26:C>T**	**2562779**	*3078953|F|0-62:C>T-62:C>T	**2646233**	C03	4116381|F|0-24:G>C-24:G>C	18167918	4338040|F|0-47:G>T-47:G>T	20237766	5.03	23.12	-1.11	-2.40	-1.29	-1.93	2.31	0.76	2.32	0.47
**C01**	**3101048|F|0-47:C>T-47:C>T**	**1713593**	**4110108|F|0-53:C>T-53:C>T**	**1644880**	C02	4166149|F|0-37:C>G-37:C>G	2621703	3145176|F|0-14:T>A-14:T>A	2632328	5.31	16.61	-3.27	-2.60	-2.32	-2.47	2.78	2.41	3.14	2.46

The logarithm of the odds (LOD) scores, additive effects (Add1 with marker1 and Add2 with marker 2 interval), Dominant (Dom 1 with marker1 and Dom2 with marker 2 intervals), Additive x dominance (Add by Dom1 and Add by Dom2 with marker1 and 2 intervals ), dominance x additive (Dom By Add1 and Dom by Add2 with marker 1 and 2 intervals ) effects and the proportion of phenotypic variance were estimated using the EPI-CIM-ADD algorithm implemented in the ICIM package. Loci detected across digenic interaction were bold (see **Table 2**). *Distance, based on cosegregating loci as linked markers did not return a significant hit. DArTseq markers were binned, and DArTseq SNPs were used to identify digenic epistatic interactions.

### 3.3 Pod length QTLs are not related to pod shattering

Previous studies showed pod shatter resistance, measured as a random impact test, correlates with pod length (Cui, 2013). To determine whether pod length variation relates to pod shattering tested with pendulum test in the F_2_ population from BC95041/BC95042, we mapped QTLs associated with pod length on A02, A05, A07, A08, A10, C02 and C05 (**Supplementary Table S6A**). Simple interval mapping identified three significant QTLs on chromosomes A05, A07, A10, and C02, whereas composite interval mapping identified two QTLs on A10 and C01 (**Supplementary Table S7A**). None of the QTLs associated with pod length was collocated with QTLs for rupture energy, suggesting that pod length is genetically not associated with rupture energy (**Table 2**, **Supplementary Table S7A**). This was further substantiated by the lack of phenotypic correlation between pod length and shatter resistance scores (*r* = 0.01, **Figure S2B**).

### 3.4 Epistatic QTL interactions modulate variation in pod shatter resistance

**Figure 5:**
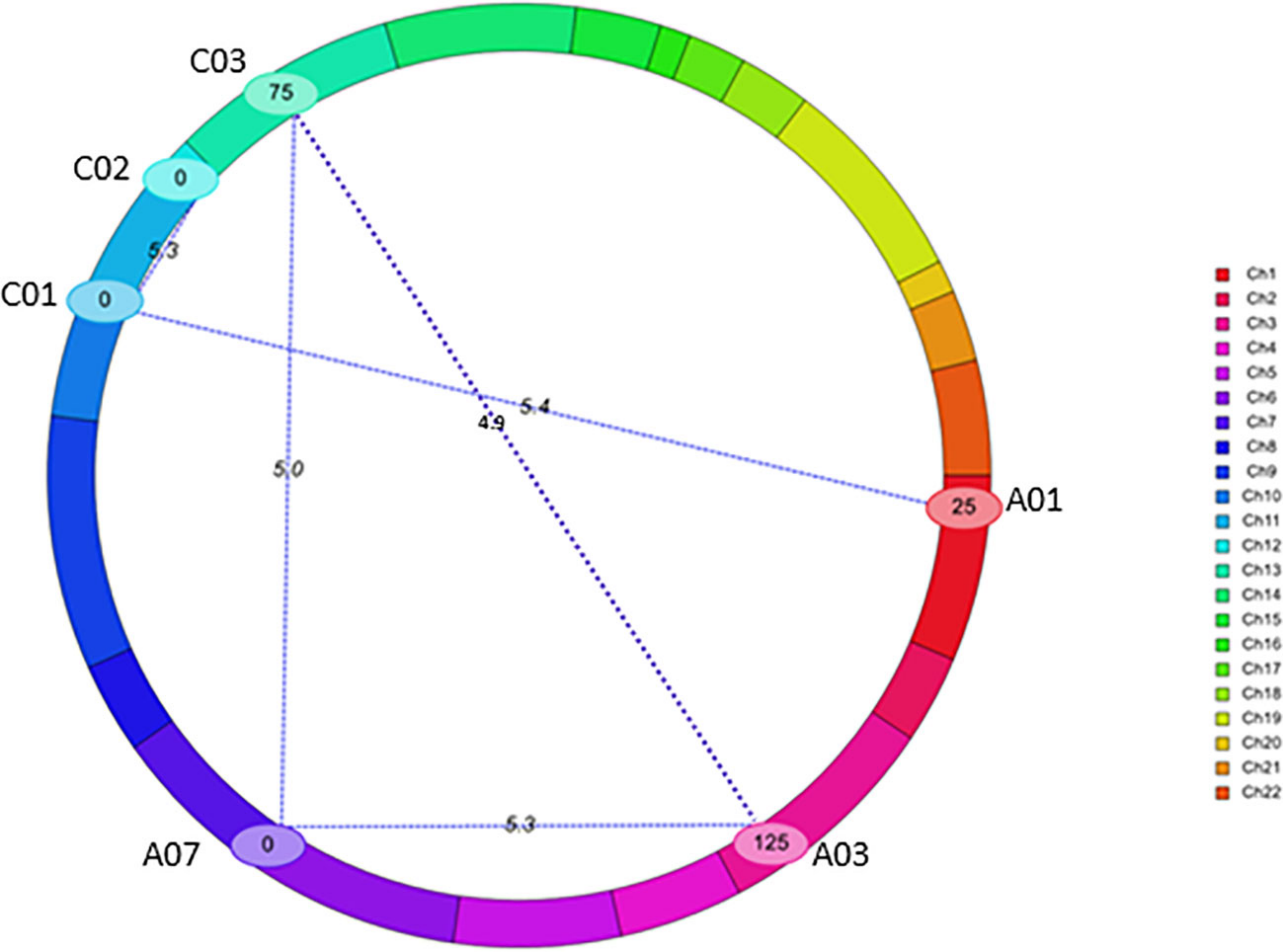
QTL interactions that showed epistatic effects for pod shatter resistance in the F2 population from BC95041/BC95042. The epistatic interaction was identified using the CIM-EPI approach in the ICIM package. Different linkage groups relating to *B. napus* chromosomes are shown (Ch1-Ch10 relate to chromosomes A1-A10, Ch11-Ch14 to C01-C04; Ch15-16 to C05, Ch17-18 to C06, Ch19 to C07, Ch20-21 to C08 and Ch22 to C09) in different colours. Interactions are shown with blue lines. Chromosomes that showed significant interactions are labelled.

Using a threshold estimated by permutation test at *P* = 0.05, 1,000 iteration (4.87), five pairs of significant epistatic QTLs for rupture energy were detected on A01/C01, A03/A07, A03/C03, A07/C03, and C01/C02 and revealed effects for additive × additive, additive × dominance and dominance × dominance interactions (**Figure 5**, **Table 3**). These EPI-QTLs accounted for 16.61% to 28.44% of PVE. Both parental alleles contributed to the epistasis in the intercross population. Additive marker effects between A03 and A07 chromosomes and A01 and C01 were in the repulsion phase. Epistatic QTLs for pod length were identified on chromosomes; A03/C07, A03/A05, A05/A08 and A05/A09, A05/C01, A05/C03, A09/C08, A09/A10, A10/C03 and A10/C08 at threshold 5 (**Table S7B**). However, using the threshold permutation test value estimated using 1,000 iterations, we did not identify any significant epistatic QTL for pod length.

### 3.5 Prioritized candidate genes underlying QTLs for pod shatter resistance

We searched for the physical location of significant markers flanking QTLs for main effects and epistatic interactions (**Tables 2**, **3**) using the *B. napus cv.* Darmor-*bzh* reference genome v4.1 (**Supplementary Tables S8A, B**). Annotated genes mapped with the QTLs marker intervals and the homologues of *priori* genes involved in pod shattering of *A. thaliana.* were inspected. Annotated genes in the reference assemblies located within QTL intervals in reference assemblies were prioritized as candidates for pod shatter resistance. The highly significant marker 3101411 associated with pod shatter resistance on A09 was mapped to the reference sequence of C08, and other cosegregating markers with 3101411 that were located at the same locus on the genetic map (16.45 cM) were mapped to the 2,177,920 to 2,443,302 bp of the Darmor-*bzh v4.1* reference sequence (**Supplementary Table S2C**). Comparative analysis identified several candidate genes, including *AP2*, *ABI3*, *ARF*, *BP1*, *CEL6*, CESA3, *FIL, FUL*, *GA2OX2*, IAA31, *IND*, *LAC4*, *LEUNIG*, *KNOTTED*, *MAGL15*, *PG1, RPL*, *QRT2*, *RGA*, *SPL* and *TCP10*) underlying main QTL and epistatic QTL interactions for pod shatter resistance. Three copies of the *FUL* gene underlie the QTLs for pod shatter resistance on chromosomes A02, A03 and A09 (**Table 2**, **Supplementary Table S10**). Marker 3129258|F|0-32:G>A-32:G>A was located 63.6 kb from BnaAnng06660D homologue of *FUL* on A02 (**Supplementary Table S9A**). The A03 QTL delimited with 5048176|F|0-11:C>T-11:C>T was mapped ~116kb apart from the *FUL* homolog (BnaA03g39820D), accounting for 19.25% % PVE. QTL on chromosome A09 delimited with 5121480|F|0-11:T>C-11:T>C marker (19.25% of the total PVE) was located near the *FUL* gene (~248Kb, BnaA09g05500D, **Table 2**). Therefore, *FUL* may contribute to genetic variation in pod shatter resistance in the population used herein. To check whether there are candidate genes that could not be retrieved based on a single reference (Darmor-*bzh* versions v4.1/10) genome assembly, we utilised the BnaOmics platform that integrates pan-genome of 26 *B. napus* reference genomes and re-sequencing data of 2,885 accessions (Cui et al., 2023). At least two *FUL* copies of A02 and A03 were located in the pan-genome (**Table S10**).

### 3.6 Sequence divergence of FRUITFULL in 373 *B. napus* varieties

*FUL* is a MADS-box transcription factor that is shown to be a part of a complex regulatory network that controls floral meristem identity, shoot maturation, floral transition, cell proliferation in pod valves and cell differentiation by limiting the dehiscence zone formation in *A. thaliana*, *B. napus* and *B. juncea* (Gu et al., 1998; Ferrandiz et al., 2000; Rajani and Sundaresan, 2001; Ostergaard et al., 2006). TBLASTN and reciprocal BLASTP searches against Arabidopsis proteins confirmed that the *FUL* (AGL8, AT5G60910) clade includes five homologues in *B. napus* on chromosomes Ann_random (BnaAnng06660D, A02 in the pan-genome), A03 (BnaA03g39820D), A09 (BnaA09g05500D), C02 (BnaC02g41870D) and C07 (BnaC07g49790D) detected in both reference genome assemblies v4.1 and 10 (**Figures S3**, **S5**). However, seven homologues were annotated in the *B. napus* pan-genome gene assembly on A02, A03, A09, C02, C07 and C09 chromosomes and validated for the presence of MADS-box domain-containing protein with a K-box coil and the MEF2 DNA-binding/dimerisation regions (**Table S10B**). *FUL* homologues of *B. napus*: BnaA03g39820D, BnaA09g0550D and BnaAnn06660D were clustered into distinct clades with *B. rapa* and BnaC02g41870D and BnaC07g49790D with *B. oleracea* clade, as expected (**Figure 2**). *FUL* homologue of *B. oleracea* (LOC10631378) showed grouping with BnaA09g0550D. Since we identified several QTLs that map near to MADS-box transcription factors such as *AGAMOUS* (*AG*), *APETALA* and *AG-LIKE* transcription factors could also regulate *FUL* expression throughout vegetative and reproductive phases during the plant development; we performed phylogenetic analysis using the Bayesian clustering method. This analysis differentiated *AG*, *FUL (AGL8)*, *SHP1* (*AGL1*), *SHP2 (AGL5)*, and *AGL3/SEPALLATA4* (*SEP4*) clades (**Figure S5**).

To date, BnaA09g05500D is the only *FUL* orthologue of *A. thaliana* and its closely related MADS-box gene in *Sinapis alba: MADSB*, which is shown to be involved in pod dehiscence via gene expression studies (Ferrandiz et al., 2000; Liiljegren et al., 2000; Chandler et al., 2005). Therefore, we further investigated its gene structure, evolution rate, and sequence variants using a dataset of 373 resequenced *B. napus* accessions utilised in the Australian National Brassica germplasm improvement program for gene discovery projects (**Table S10**). To determine the gene structure of the *FUL homologues* in *B. napus*, we used *AtFUL* (AT5G60910, TAIR). Sequence analysis of BnaA09g0550D) revealed that it encodes a 726 bp transcript with a 242 amino acid protein and comprises 8 exons and 7 introns (http://www.genoscopegen.cns.fr/brassicanapus/cgi-bin/geneView?src=colza;name=BnaA09g55330D) (**Figure S3**). The size of the first intron (intron 1) varied from 861 (A02) to 2462 (C02) bp, in contrast to some plant species, such as tomato and the wild D-genome progenitor of bread wheat, *Aegilops tauschii* (Takumi et al., 2009; Maheepala et al., 2019). The parental lines of the mapping population from BC95041 and BC95042 revealed 364 polymorphic SNPs and deletions NCBI, Banklt accession ID 2735083, (**Table S11**), and two non-synonymous variants were identified in exon 1 (c.A25G:p.K9E) and 7 (c.G616T:p.A206S). There were five non-synonymous SNV in exon 1 (c.G166A:p.E56K, c.G155A:p.G52D, c.G139A:p.V47I and c.A25G:p.K9E) and exon 7 (c.G616T:p.A206S) of BnaA09g05500D. Among all 373 accessions, up to 578 variants were detected in *FUL* homologues in *B. napus*; the majority (~50%) occurred in the intergenic region, followed by intronic regions (**Table S11**). Sequence variants were detected in the exonic and upstream sequence of *FUL* homologues, ranging from 19 to 36 and 11-99, respectively. We also identified splice variants for BnaA09g05500D (1 variant) and BnaAnng06660D gene (2 variants).

We performed selection pressure analysis to determine the evolution rate as the ratio of Ka/Ks of *FUL* copies. Our results show that BnaA09g05500D copy on chromosome A09 had purifying selection (<0.1) followed by copies on C02, suggesting conserved function compared to BnaA03g39820D and BnaC07g49790D on A03 and C07, respectively (**Supplementary Table S10C**).

**Figure 6:**
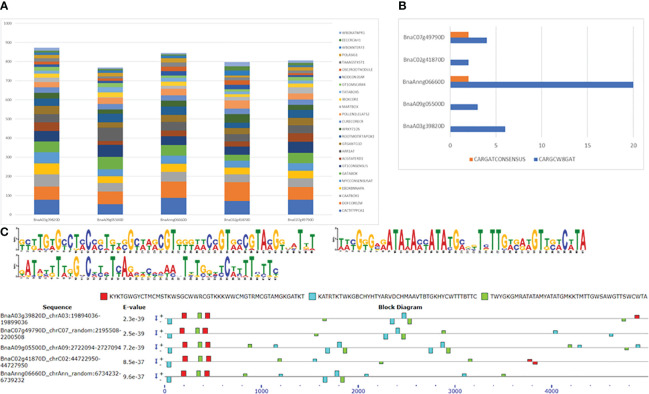
Motif identification in 5 Kb upstream sequences of five *FUL* homologues in *B. napus*. **(A)** Duplication frequency of 25 most abundant motifs. **(B)** CArG motifs and their frequency of occurrence. **(C)** Three novel motifs found using MEME and their occurrence in the sequence. + and – indicates the motif occurrence on sense and antisense strands.

Analysis of 5 kb upstream regions of five *FUL* homologues with the SIGNALSCAN program within the PLACE database (https://www.dna.affrc.go.jp/PLACE/?action=newplace) revealed several motifs found in plant cis-acting regulatory DNA elements. The search identified 183 motifs, ranging from 127 in BnaC02g41870D to 145 in BnaA03g39820D). Of these 183 motifs, 91 common motifs were present in all five homologues, while 25 were unique to one of them. The duplication frequency of these common motifs in all five genes is depicted in **Figure 6A**, and the numbers are given in **Table S12**. Among the common motifs, DOFCOREZM is the most abundant one, with duplication frequency of 66 to 98 in the 5 Kb upstream region of *FUL* homologues, followed by CACTFTPPCA1, GT1CONSENSUS GATABOX and CAATBOX1. The *FUL* gene is shown to bind to a specific CArG box, with the consensus sequence CC(A/T)6GG (de Folter and GC, 2006). In *B. napus*, 2 to 20 CArG motifs (CARGCW8GAT and CARGATCONSENSUS) were found in the upstream sequence of *FUL* homologs. We identified CArG consensus sequence (CCWWWWWWGG) in BnaAnng06660D and BnaC07g49790D only, whereas a variant of CArG motif with a more extended A/T-rich core (CWWWWWWWWG) is found in upstream sequences of all five *FUL* homologues (**Figure 6B**). There were 14 motifs (ABRELATERD1, ACGTATERD1, ACGTABREMOTIFA2OSEM, CBFHV, DRECRTCOREAT, LTRECOREATCOR15, MYB1AT, MYB2AT, MYBATRD22, MYBCORE, MYB2CONSENSUSAT, MYCCONSENSUSAT, MYCATERD1 and MYCATRD22) detected in the dataset which are associated with water stress or dehydration. Consistent with previous studies, we also found auxin response elements (GGTCCCATGMSAUR, AUXREPSIAA4, AUXRETGA1GMGH3, ARFAT, SURECOREATSULTR11 and CATATGGMSAUR) in our upstream sequences dataset. Among these motifs, SURECOREATSULTR11 and CATATGGMSAUR were found in the upstream sequences of all five genes, whereas GGTCCCATGMSAUR and AUXREPSIAA4 were unique to the upstream sequence of BnaA03g39820D (**Table S12**). Furthermore, seven motifs (WRKY71OS, PYRIMIDINEBOXOSRAMY1A, PYRIMIDINEBOXHVEPB1, GAREAT, MYBGAHV, GADOWNAT and GARE2OSREP1) were associated with gibberellin signalling pathway. The chromosome A09 *FUL* copy also had the maximum number (14) of SAUR (Small Auxin-Up RNA, CATATGGMSAUR) motifs, implicated in auxin responsiveness (Xu and Guilfoyle, 1997). Copy number variation and distribution of motifs in the upstream regulatory region of *FUL* may account for natural variation in gene expression and regulation of valve growth by interacting with other genes involved in valve margin differentiation, such as *SHP1*, *SHP2*, *IND* and *ALC*. *IND* also forms auxin minimum by coordinating auxin efflux in separation layer cells (Sorefan et al., 2009). We also found the GTGANTG10 motif (with duplication frequency 28-43), which shows homology to pectate lyase (Rogers et al., 2001).

We also discovered three unknown motifs in the 5 Kb upstream sequences of all five *FUL* homologues. The first motif KYKTGWGYCTMCMSTKWSGCWWRCGTKKKWWCMGTRMCGTAMGKGATKT (GCGTGTGCCTCCCCTGTCGCAAGCGTGGGAACCGTGCCGTACGGGATGT) is potentially located within first 500bp upstream, whereas the second motif KATRTKTWKGBCHYHTYARVDCHMAAVTBTGKHYCWTTTBTTC (GATGCGTTGGCCCCCTCAGCGCCCAACTGTGGCCCATTTCTTC) and the third motif TWYGKGMRATATAMYATATGMKKTMTTGWSAWGTTSWCWTA (TACGGGCGATATACCATATGCGGTCTTGACAAGTTCACATA) are randomly dispersed with no particular pattern detected in their occurrence with respect to positions (**Figure 6C**). Also, the first motif is mainly detected on the sense strand, whereas the second and third motifs are comparatively present on both sense and antisense strands.

## 4 Discussion

Seed shattering is a massive issue in commercial canola production worldwide, underpinning growers’ profitability. Pod shatter-resistant varieties suitable for direct harvesting with combines are essential to reduce (i) reliance on windrowing, (ii) yield losses, (iii) inputs cost (labour and fuel for windrowing and controlling rogues in subsequent crops), (iv) carbon emissions occurred while windrowing followed by threshing with combine harvesters, and to improve (v) gross margins of farmers (return on the investment).

Herein, we investigated the genetic basis of pod shatter resistance in an interspecific derivative of *B. rapa*/*B. napus*. In this study, we used the pendulum test to describe the genetic variation for pod shatter resistance in a quantitative manner and understand its underlying genetic and anatomical bases. Previously, several methods, such as the number of seeds lost from pods, the number of seedlings germinated, the random impact test, and the pendulum test, have been used to determine genetic variation for pod shatter resistance in *Brassica* species (Morgan et al., 1998). There were 6.23-fold differences in pod shatter resistance between parental lines, suggesting that the interspecific source, BC95042, could be used to improve resistance to pod shatter.

Genetic analysis showed that pod shatter resistance is due to seven QTLs located on A02, A03, A05, A09 and C01 chromosomes in an F_2_ population derived from a cross between BC95041 and BC95042 (**Table 2**). With linear marker regression, HTR, IM, and CIM algorithms, we repeatedly detected four QTLs for pod shatter resistance on A02, A05 and A09, suggesting these QTLs are reliable for research and development activities such as introducing appropriate favourable alleles into canola varieties. Using different mapping algorithms with robust statistical power ensured the identification of significant marker-trait associations by reducing false positives to make genetic gains in canola breeding programs. Previous genetic mapping studies identified QTLs for pod shatter resistance in *B. rapa* (Mongkolporn et al., 2003; Bagheri et al., 2012), *B. juncea* (Kaur et al., 2020) and *B. napus* (Hu et al., 2012; Wen et al., 2013; Raman et al., 2014; Liu et al., 2016). Some of the QTLs were located in similar genetic positions on *B. napus* genome, which were detected in earlier studies (**Table S9**). However, there were no overlapping QTL regions across populations of Chinese origin. For example, Liu et al. (2016)) reported six significant QTLs for pod shatter resistance in a *B. napus* GWAS panel and two structured biparental populations on A01, A06, A07, A09, C02, and C05 chromosomes. Two QTLs on A06 and A09 were repeatedly detected across environments and mapping panels. QTL on A09 delimited with an Illumina SNP marker, Bn-A09-p30171993, was mapped near the *SHP1* gene (A09_random chromosome on the 4.1 Darmor-*bzh* assembly). However, *SHP1* and Bn-A09-p30171993 were located at the distal end of the A09 chromosome (Darmor-*bzh* version 10). However, this study identified three QTLs on chromosomes A02, A03 and A09 that significantly contributed to pod shatter resistance, accounting for 9.42% and 19.25% of the total PVE, respectively, and map near the *FUL* homologues (BnaAnng06660D, BnaA03g39820D and BnaA09g05500D, **Table 1**). These QTLs were not detected in other *B. napus* populations (Wen et al., 2013; Liu et al., 2016). We could not compare the map position of 13 QTLs for pod shatter resistance, measured by improved random impact method on A01, A04, A07, A08, C05, and C08 (Wen et al., 2013) as they were not mapped on any physical map of *B. napus*. Our study did not detect any QTL on A06 for pod shatter resistance located near the *GIBBERELLEIN-3-OXIDASE1* gene in *B. napus* populations of Chinese origin (Liu et al., 2016). Most QTLs on A01, C02, and C05 were not closely mapped. These observations hint that selection for pod-shattering may have occurred at several independent loci and shaped the genomic architecture of pod-shatter resistance during cultivation and selective breeding in *B. napus*. This hypothesis is supported by independent seed-shattering QTLs (on A03, A09, this study) and the absence of the *SHP1* and *TCP8* genes, as shown in earlier studies (Liu et al., 2016; Liu et al., 2020; Chu et al., 2022). During domestication, Brassica species may have acquired several shattering resistance mechanisms to reach the desirable level of shattering resistance, suitable for manual harvesting, probably under humid climates, e.g., Europe and Wuhan. However, the resistance level is insufficient for hot and dry climates, e.g., Australia.

The PVE (6.29 to 20.80%) and additive effects from both parental lines (-4.28 to 1.78) that we identified in this study were consistent with most of the published *B. napus* studies revealing a small to moderate proportion of genotypic variation (4.01 to 28.9%) in pod shatter resistance (Wen et al., 2013; Raman et al., 2014; Liu et al., 2016; Liu et al., 2020). A recent study shows a major gene (i.e. *TCP8* on C09) effect on pod shatter resistance via a lignified-layer bridge in a *B. napus* population (Chu et al., 2022). Our digenic interaction analysis showed five epistatic QTL interactions between chromosomes (A01-C01, A03-A07, A07-C03, A03-C03, and C01-C02). The positive epistatic effect of additive x additive suggested that the two epistatic loci (e.g. A03/C03, A07/C03, and C01/C02) with homozygous/heterozygous alleles from the same parent could increase the pod shatter resistance. However, the positive additive × dominance epistatic effect indicated that BC95042 could increase the pod shatter resistance. Breeding programs must consider additive and additive x additive epistatic interactions to improve resistance to pod shatter.

**Figure 7:**
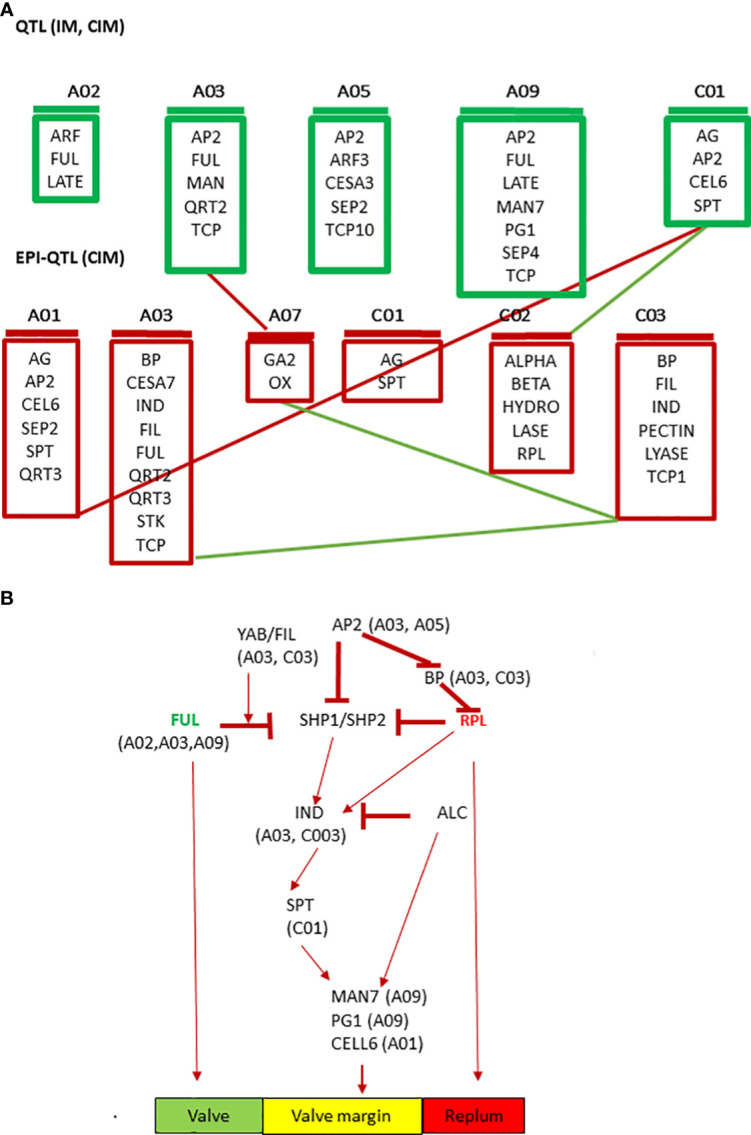
Prioritized candidate genes underlying QTL for pod shatter resistance using the simple interval, composite interval mapping, and epistatic-composite interval mapping algorithms implemented in the ICIM package. **(A)** Cartoon showing QTL with main effects (IM, CIM) and epistatic interactions (epi-QTL) along with their chromosomal location and **(B)** Extrapolated QTL-based candidate genes involved in pod shatter resistance network in *B. napus.* The green colour indicates valve, the yellow colour valve margin identity-related genes, and the orange colour indicates the replum.

Based on the physical location of linked markers associated with pod shatter resistance, we prioritized *AG*, *ABI3*, *ARF3*, *BP1*, *CEL6*, *FIL*, *FUL*, *GA2OX2*, *IND*, *LATE*, *LEUNIG*, *MAGL15*, *RPL*, *QRT2*, *RGA*, *SPT* and *TCP10*, as candidate genes for pod shatter resistance (**Table S9**). The mechanisms and genetic factors involved in pod dehiscence have been investigated in *A*. *thaliana* and its closely related Brassica species. MADX-box transcription factors encoding *FUL*, *SHP1*, and *SHP2* are the major players that control fruit patterning, lignin deposition, and pod dehiscence in Arabidopsis (Gu et al., 1998; Liiljegren et al., 2000). *FUL* negatively regulates *SHP* and *IND* expression in the valve margin and *APETALA 1* in the outer whorl of the flower (Ferrandiz et al., 2000; Kaufmann et al., 2010). *FUL* and BEL-subfamily homeodomain gene *RPL* also negatively regulate *SHP* expression in the valve margin (Roeder et al., 2003). The floral homeotic gene *AP2* also negatively regulates the expression of *SHP*, *RPL*, and *IND* genes and the expansion of replum and lignified layers (Ripoll et al., 2011). *SHP1* and *SHP2*, which act redundantly, regulate the expression of basic helix-loop-helix (bHLH) genes: *ALC*, *IND*, and *SPATULA* (*SPT*). *SHP1/2* and *IND* cause pod dehiscence by promoting cell proliferation and are involved in the differentiation of the lignification and separation layers in the stripes of the valve margin, whereas *ALC* and *SPT* are involved in forming the separation layer (Rajani and Sundaresan, 2001; Liljegren et al., 2004; Lewis et al., 2006; Groszmann et al., 2011). *IND* activates the expression of *ALC* and *SPT* but also promotes its own heterodimerisation with them through DELLA protein degradation (Girin et al., 2010; Girin et al., 2011). Finally, *ALC* and *SPT* are able to repress *IND* expression (Lenser and Theissen, 2013). *IND* regulates gibberellin levels through the *GA3 Oxidase 1/GA4* gene (Arnaud et al., 2010; Kay et al., 2013). *FIL*, *YABBY* and *JAG* can control the expression patterns of *FUL* and *SHP* in the valve and valve margins ( (Dinneny and Yanofsky, 2005; Mühlhausen et al., 2013). We also identified downstream genes such as *BETA-1-4 GLUCANASE* (*CELLULASE6*), ENDO-POLYGALACTURONASE (*RDPG1*, *QRT2*), *MAN7*, *NST1/3* and other MADS family transcription factors like *SEPALLATA3*, *AGL15, SEP4*, associated with pod shatter resistance in the mapping population. These genes are implicated in pod dehiscence in *A. thaliana* and *B. napus* (Jiang et al., 2016; Li et al., 2021). Di Marzo et al. (2022), found that the expression of α-XYLOSIDASE1 (*XYL1*) is directly regulated in developing seeds and fruit by the MADS-box transcription factor *SEEDSTICK* (*STK*). They demonstrated that *XYL1* complement the *stk* smaller seed phenotype, confirming the importance of cell wall modulation in shaping organs. Some *priori* genes for pod shatter resistance were localised more than 1Mb from significant QTL regions. Small populations with low-density markers cannot resolve recombination between markers and candidate genes (Raman et al., 2016). However, the homologs of pod shatter resistance genes that map further apart from significantly associated markers on other chromosomes could regulate genetic variation in pod shatter resistance. Further research is required to substantiate this hypothesis. We identified sequence variants between the parental lines of the mapping population and other elite lines of *B. napus*. Further studies are required to establish the role of sequence variants in pod shatter resistance genes and their functional role via gene expression and gene editing approaches. Overall, our data on genetic mapping and putative candidate/priority genes suggest the complex network involved in pod shatter resistance in *B. napus* germplasm, broadly consistent with *A. thaliana* (**Figure 7**), as reiterated earlier (Stephenson et al., 2019). This observation is consistent with the high syntenic relationships between *B. napus* and *A. thaliana* (Parkin et al., 2005).

In summary, we constructed the genetic framework map and identified seven genomic regions associated with pod rupture energy on A02, A03, A05, A09, and C01 chromosomes in an F_2_ population derived from the BC95041/BC95042 line developed from *B. rapa*/*B. napus*. In addition, five pairs of significant epistatic QTL interactions for rupture energy between A01/C01, A03/A07, A07/C03, A03/C03, and C01/C02 chromosomes. Overall, our results showed that independent QTLs (on A02, A03, A05, A09 and C01 chromosomes) and interactive QTLs (on A01/C01, A03/A07, A07/C03, A03/C03, and C01/C02) contribute to genetic variation in pod shatter resistance. Epistatic QTL interactions possibly reflect the regulatory network (repressor and activators) involved in pod dehiscence in *A. thaliana.* Several QTL regions were mapped near the candidate genes (*AG, ABI3, ARF3, BP1, CEL6, FIL, FUL, GA2OX2, IND, LATE, LEUNIG, MAGL15, RPL, QRT2, RGA, SPT*, and *TCP10*) which are involved in pod dehiscence, primarily in Arabidopsis. We described putative *cis*-acting motifs and sequence variants in genic and promoter regions of *FUL* homologues in 373 *B. napus* accessions. This study provides a valuable resource for gene discovery, the molecular mechanism underlying pod shatter resistance and yield improvement in *Brassica* species. DNA markers could accelerate the use of QTL in the Brassica breeding programs for marker-assisted selection, backcross, and genomic selection pipelines.

## 5 Conclusions

This study found that the interspecific line, BC94052 has superior alleles for resistance to pod shatter. Our genetic mapping suggests pod shatter resistance is due to multiple loci; three QTLs map to the A02, A03 and A09 chromosomes near *FUL* homologues. Our research provides a valuable genetic resource for improving pod shatter resistance in canola and for future studies on understanding molecular mechanisms underlying pod shatter resistance.

## Data availability statement

The datasets presented in this study can be found in online repositories. The Illumima sequence data of FULL genes can be found in NCBI Banklt accesssion 2735083.

## Ethics statement

The authors declare that the experiments comply with the current laws of the country in which they were performed and comply with ethical standards.

## Author contributions

HR and RR designed the research and analyzed the data. RR developed the mapping population and conducted the experiments. YQ and BM assisted in phenotyping and performed pod anatomy and DNA extractions. NS, XC, YZ, QH, HR, and SL aligned DArTseq data with the reference genomes and analysed the dataset. NG provided the seeds of an interspecific line. HR wrote the first draft, NS contributed to the sections and all authors approved the final draft of the manuscript.
