# Supplementary material for: Novel quantitative trait loci from an interspecific Brassica rapa derivative improve pod shatter resistance in Brassica napus
Source: Front Plant Sci. 2023 Sep 6;14:1233996. doi: 10.3389/fpls.2023.1233996 (PMC10510201; doi:10.3389/fpls.2023.1233996)
Supplement: Supplementary file 1 [file DataSheet_1.zip › Figures S1-6.pptx]

## Slide 1
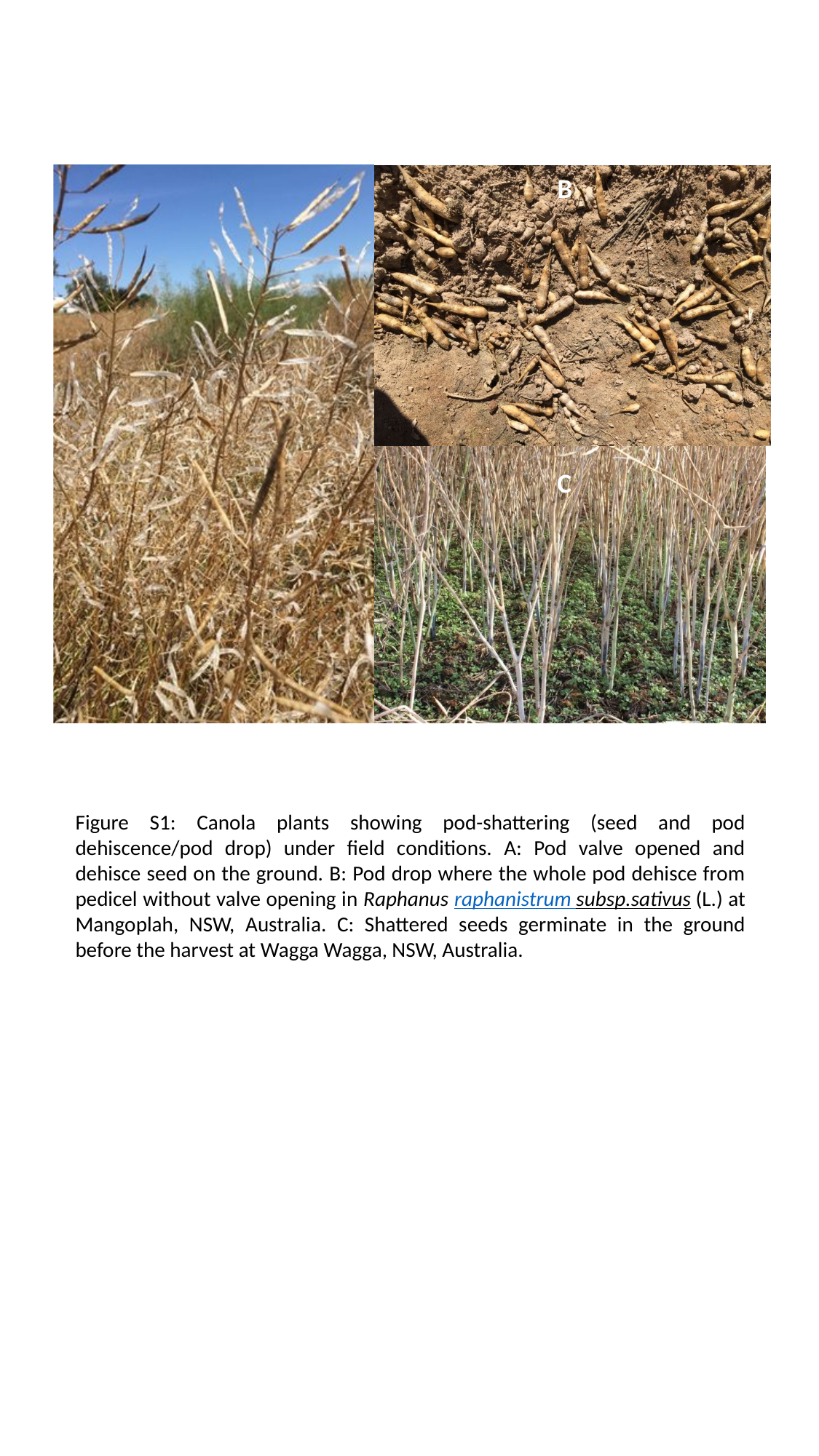

A						B
						C
Figure S1: Canola plants showing pod-shattering (seed and pod dehiscence/pod drop) under field conditions. A: Pod valve opened and dehisce seed on the ground. B: Pod drop where the whole pod dehisce from pedicel without valve opening in Raphanus raphanistrum subsp.sativus (L.) at Mangoplah, NSW, Australia. C: Shattered seeds germinate in the ground before the harvest at Wagga Wagga, NSW, Australia.

## Slide 2
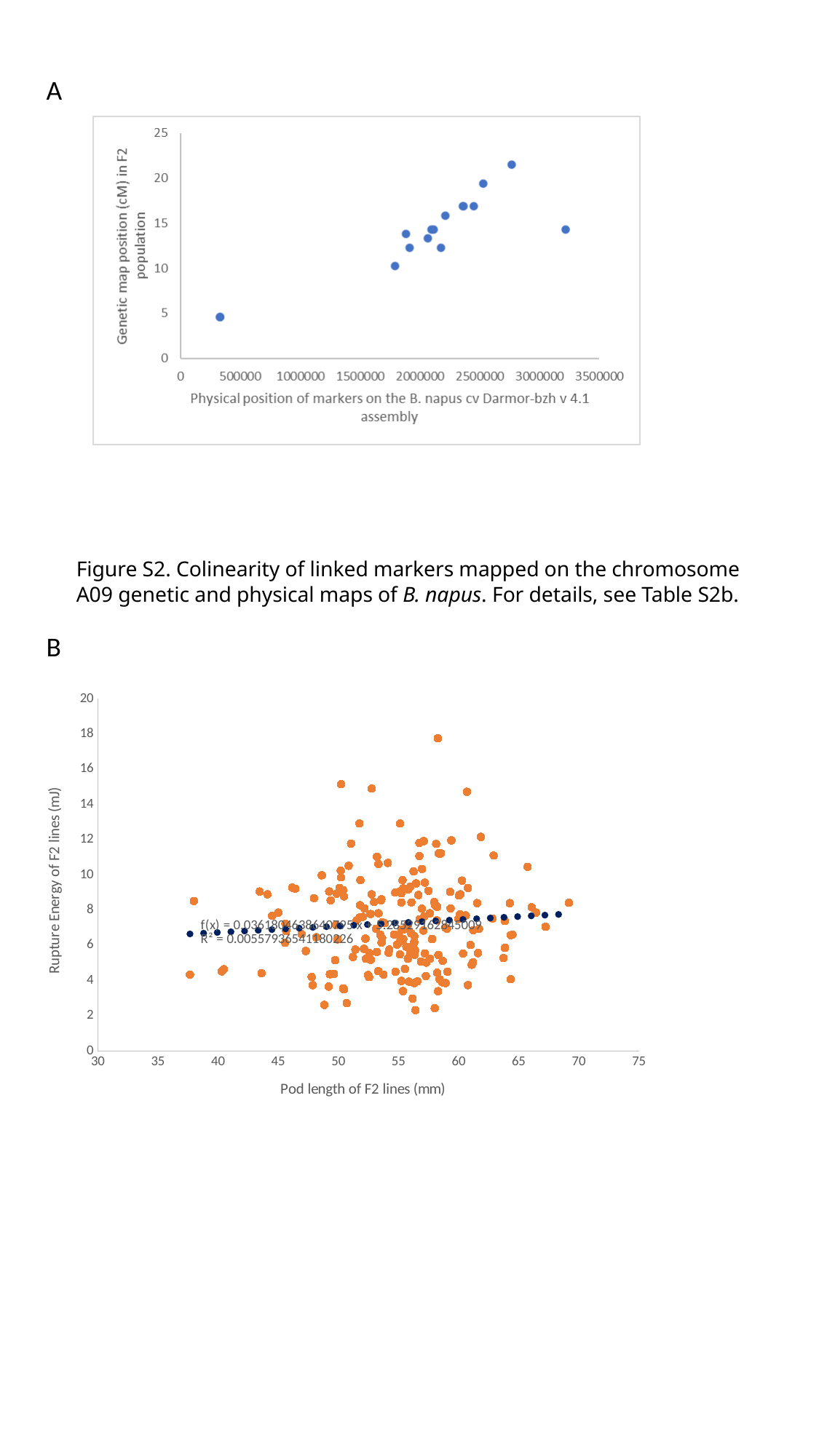

A
B
# Figure S2. Colinearity of linked markers mapped on the chromosome A09 genetic and physical maps of B. napus. For details, see Table S2b.
### Chart
| Category | |
|---|---|

## Slide 3
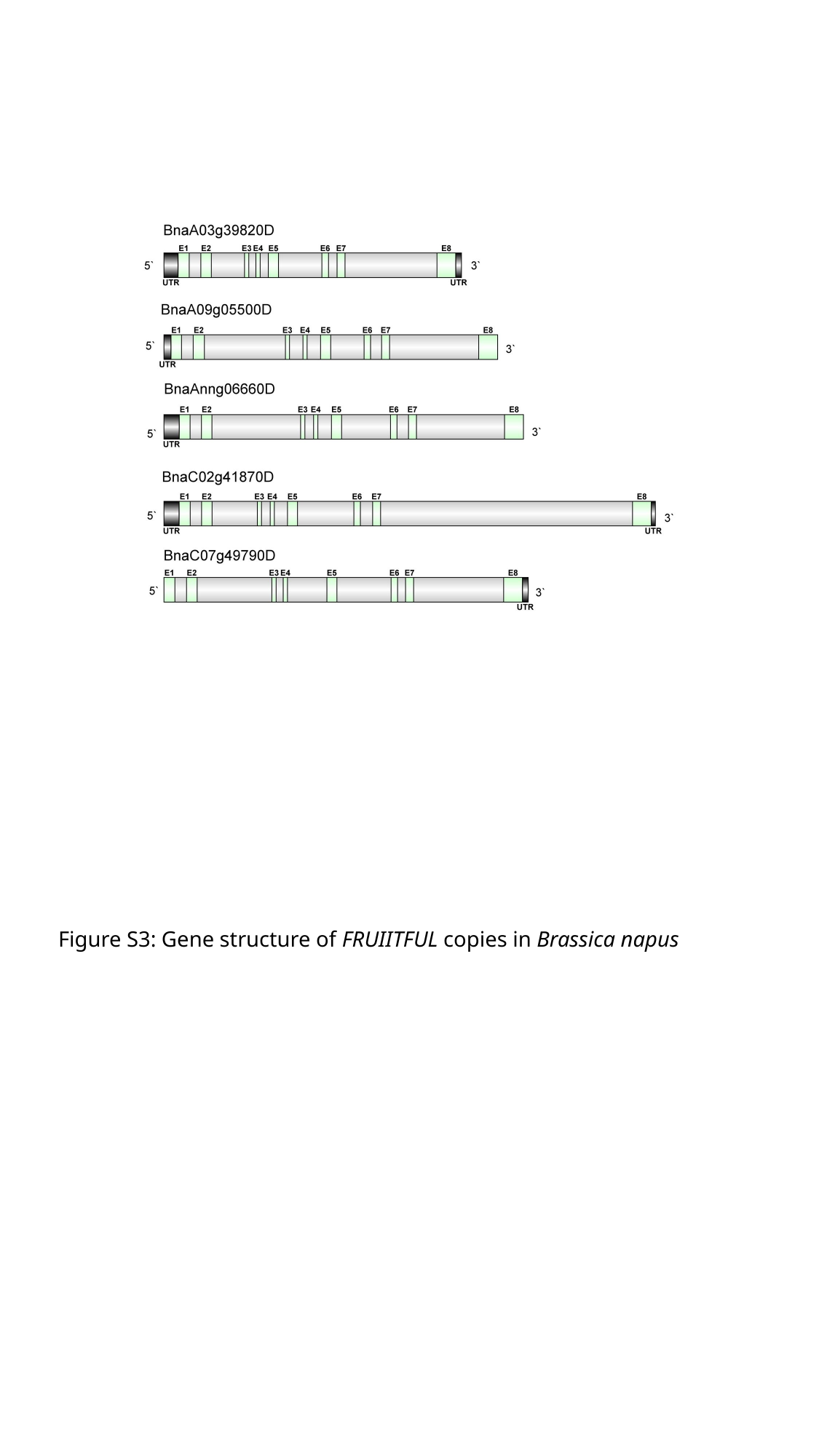

# Figure S3: Gene structure of FRUIITFUL copies in Brassica napus

## Slide 4
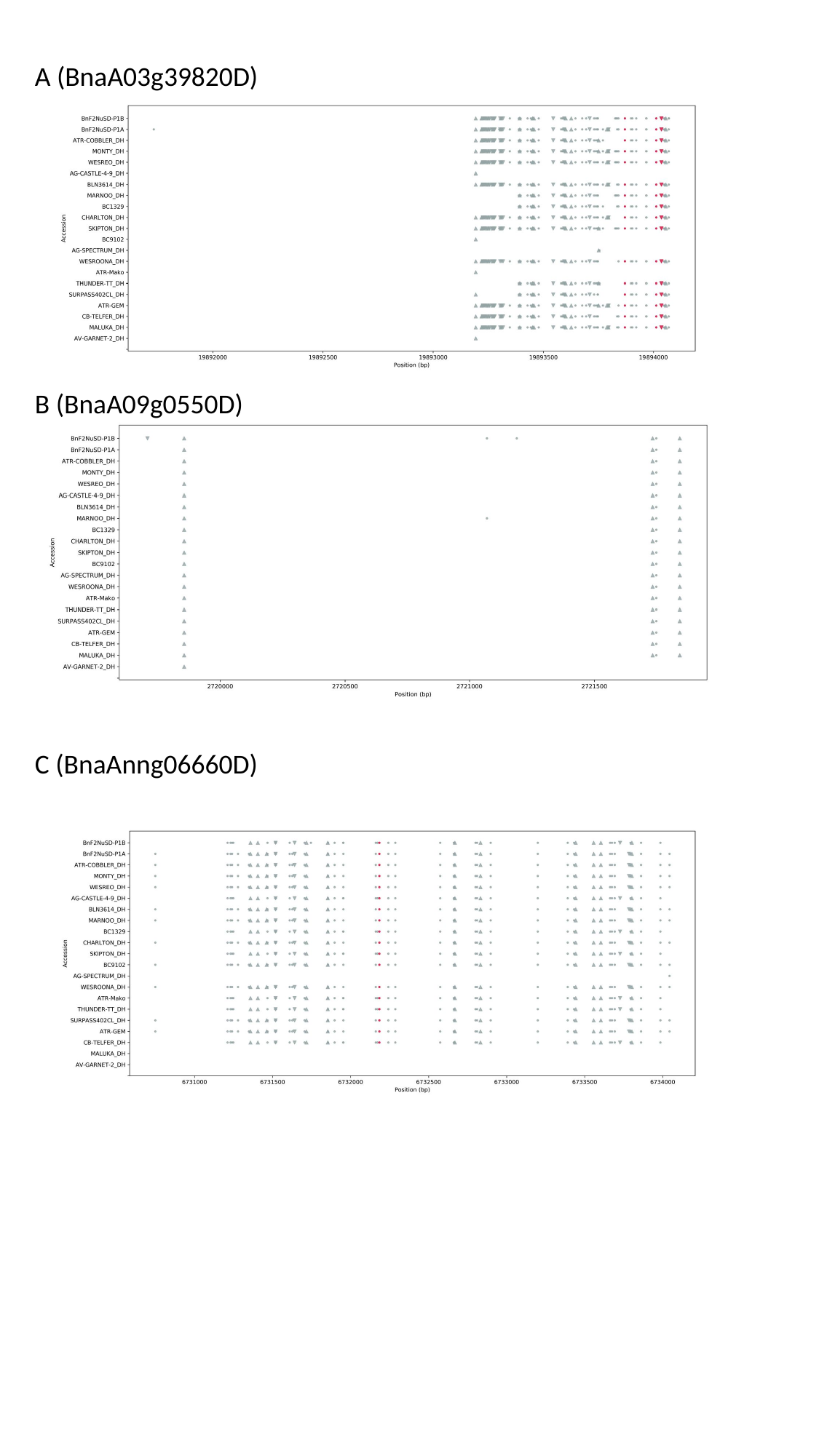

A (BnaA03g39820D)
B (BnaA09g0550D)
C (BnaAnng06660D)

## Slide 5
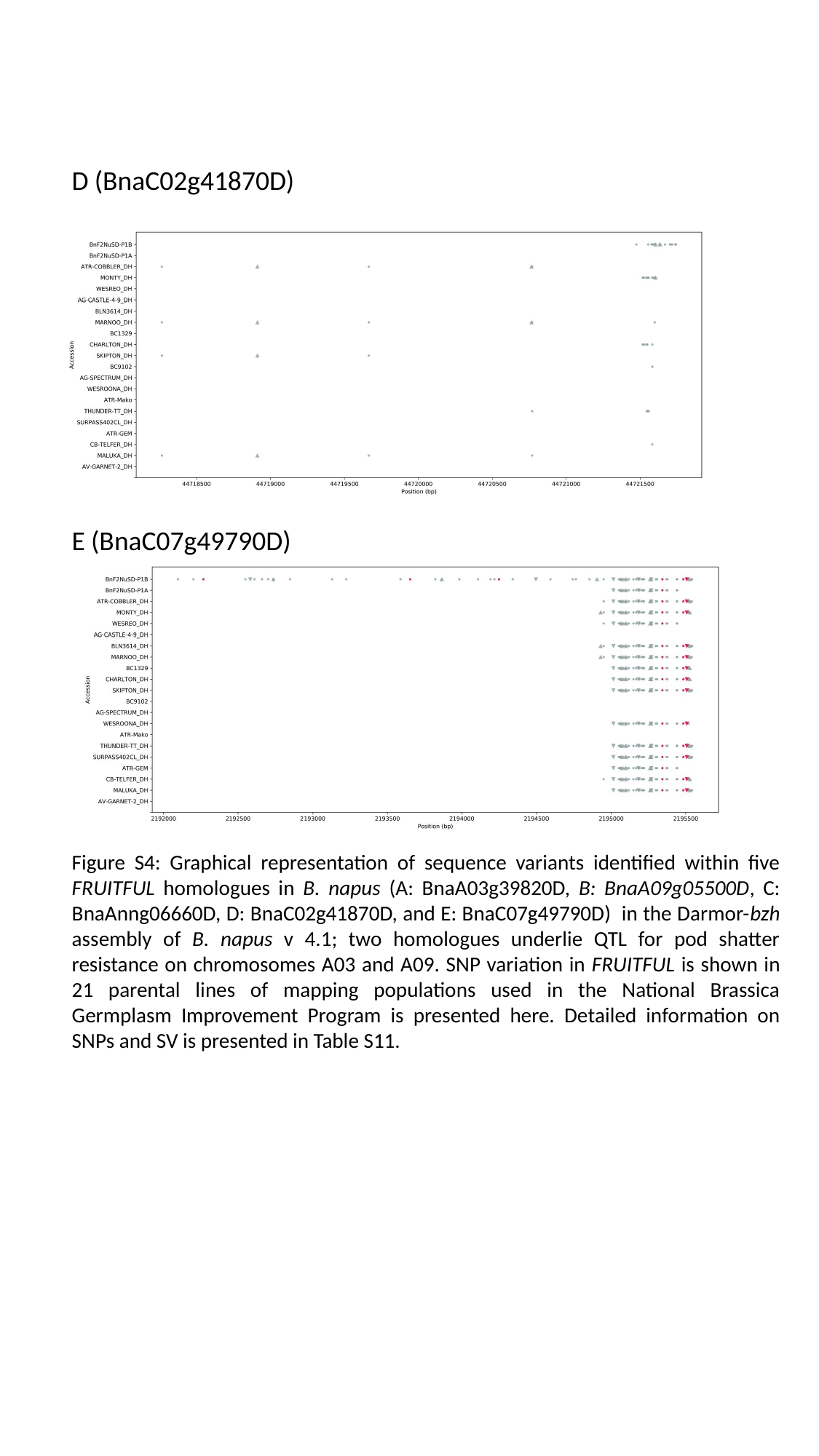

D (BnaC02g41870D)
E (BnaC07g49790D)
Figure S4: Graphical representation of sequence variants identified within five FRUITFUL homologues in B. napus (A: BnaA03g39820D, B: BnaA09g05500D, C: BnaAnng06660D, D: BnaC02g41870D, and E: BnaC07g49790D) in the Darmor-bzh assembly of B. napus v 4.1; two homologues underlie QTL for pod shatter resistance on chromosomes A03 and A09. SNP variation in FRUITFUL is shown in 21 parental lines of mapping populations used in the National Brassica Germplasm Improvement Program is presented here. Detailed information on SNPs and SV is presented in Table S11.

## Slide 6
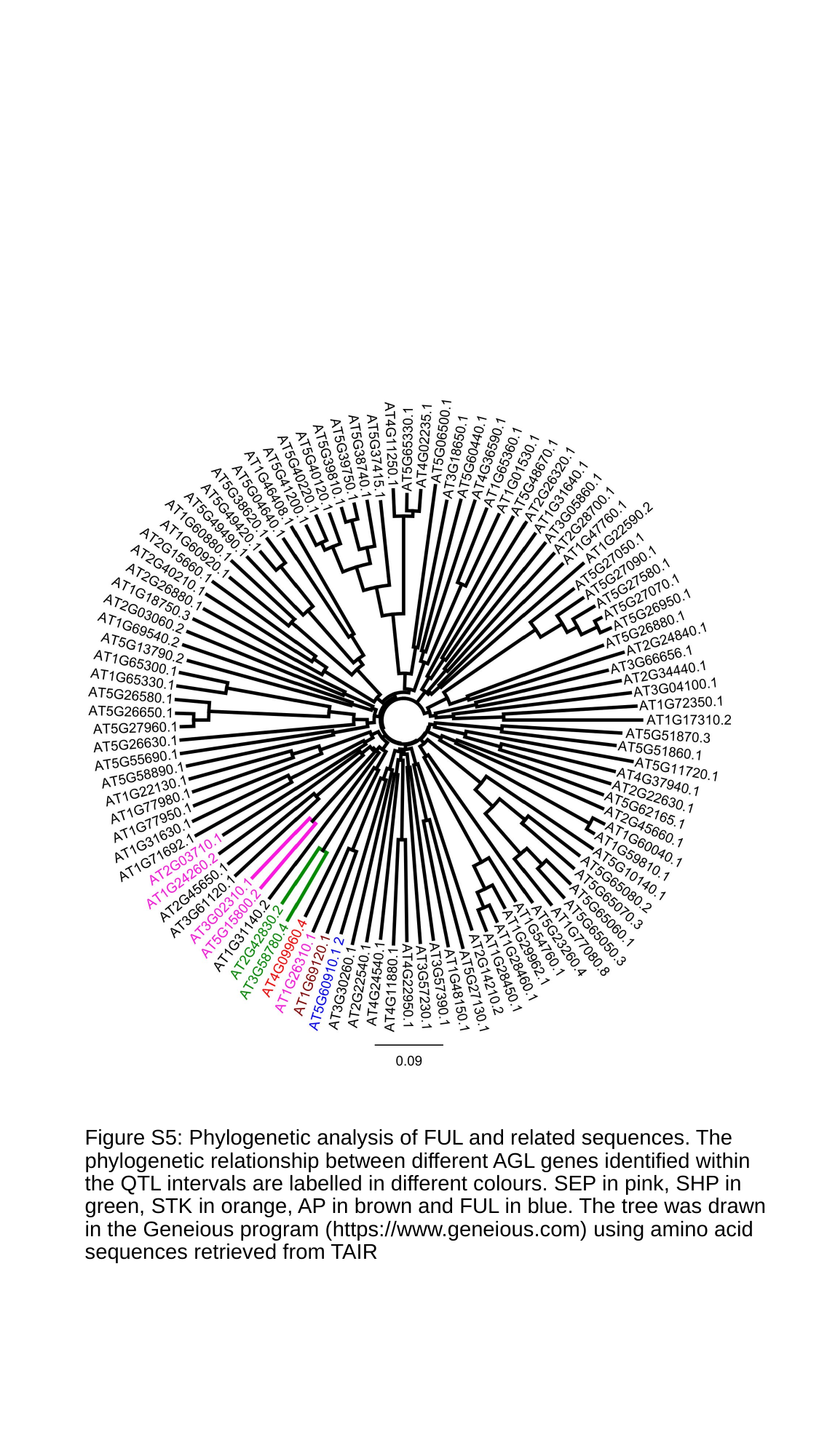

# Figure S5: Phylogenetic analysis of FUL and related sequences. The phylogenetic relationship between different AGL genes identified within the QTL intervals are labelled in different colours. SEP in pink, SHP in green, STK in orange, AP in brown and FUL in blue. The tree was drawn in the Geneious program (https://www.geneious.com) using amino acid sequences retrieved from TAIR

## Slide 7
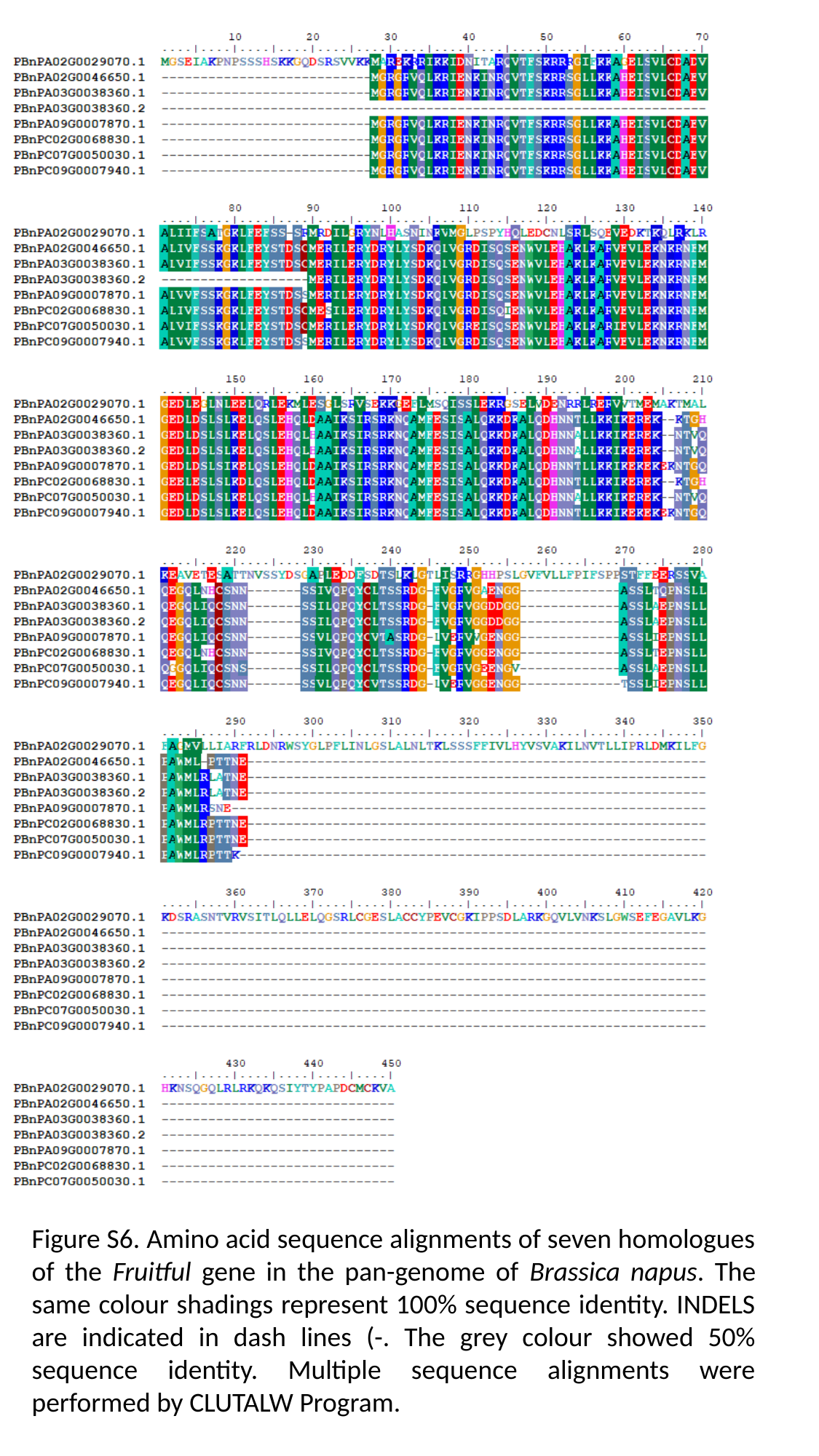

Figure S6. Amino acid sequence alignments of seven homologues of the Fruitful gene in the pan-genome of Brassica napus. The same colour shadings represent 100% sequence identity. INDELS are indicated in dash lines (-. The grey colour showed 50% sequence identity. Multiple sequence alignments were performed by CLUTALW Program.
